# Heart rate processing algorithms and exercise duration on reliability and validity decisions in biceps-worn Polar Verity Sense and OH1 wearables

**DOI:** 10.1038/s41598-023-38329-w

**Published:** 2023-07-20

**Authors:** James W. Navalta, Dustin W. Davis, Elias M. Malek, Bryson Carrier, Nathaniel G. Bodell, Jacob W. Manning, Jeffrey Cowley, Merrill Funk, Marcus M. Lawrence, Mark DeBeliso

**Affiliations:** 1 Department of Kinesiology and Nutrition Sciences, University of Nevada, Las Vegas, Las Vegas, NV USA; 2 Interdisciplinary Health Sciences, University of Nevada, Las Vegas, Las Vegas, NV USA; 3 Department of Kinesiology, California State University, San Bernardino, San Bernardino, CA USA; 4 Department of Kinesiology and Outdoor Recreation, Southern Utah University, Cedar City, UT USA

**Keywords:** Physiology, Health occupations, Translational research

## Abstract

Consumer wearable technology use is widespread and there is a need to validate measures obtained in uncontrolled settings. Because no standard exists for the treatment of heart rate data during exercise, the effect of different approaches on reliability (Coefficient of Variation [CV], Intraclass Correlation Coefficient [ICC]) and validity (Mean Absolute Percent Error [MAPE], Lin’s Concordance Correlation Coefficient [CCC)] were determined in the Polar Verity Sense and OH1 during trail running. The Verity Sense met the reliability (CV < 5%, ICC > 0.7) and validity thresholds (MAPE < 5%, CCC > 0.9) in all cases. The OH1 met reliability thresholds in all cases except entire session average (ICC = 0.57). The OH1 met the validity MAPE threshold in all cases (3.3–4.1%), but not CCC (0.6–0.86). Despite various heart rate data processing methods, the approach may not affect reliability and validity interpretation provided adequate data points are obtained. It is also possible that a large volume of data will artificially inflate metrics.

## Introduction

Heart rate (HR) is used as a physiological indicator of exercise intensity by athletes, coaches, and recreational exercisers^1^. Many exercise prescriptions are based on heart rate range, either as a percent of maximal^2^ or using a relative level such as with the Karvonen formula^3^. It becomes important then for individuals to accurately obtain heart rate during exercise and physical activity. Wearable technology has become nearly universally utilized^4^. These wearable devices return a variety of metrics including step count^5^, energy expenditure^6^, and heart rate^7^. Wearable devices have been used to provide metrics for many public health issues. For example, heart rate measurements can be incorporated into artificial pancreas systems to improve glycemic control, serving as a useful tool for managing diabetes^8,9^. Moreover, wearable devices can be used to track and monitor stress management^10^, obesity^11^, heart failure^12^, sleep disorders^13^, and cardiovascular disease^14^. Therefore, accurate wearable devices have the potential to improve the outcomes of a wide range of public health concerns. Investigating the reliability and validity of different wearable devices provides valuable information.

When considering the variable of heart rate during exercise, wearable technology investigations have used a variety of processing algorithms to evaluate the concurrent validity of wearable devices against criterion devices. Some studies have used a cross-sectional approach, obtaining a single HR measurement at specific intervals such as one measure every second^7,15–23^, 15 s^24^, 30 s^25^, or 60 s^25–30^. Other investigations have processed the heart rate data by taking an arithmetic mean over specific intervals, including 5-s epochs^31–34^, 10-s epochs^35^, the exercise stage during steady state activities of differing intensity^36^, or the entire bout^37^. It is unknown what effect differences in the data processing of heart rate may have on the ultimate decision of agreement, validity, and reliability in wearable devices.

Another unanswered question is what effect the exercise duration has on decisions of validity and reliability. Our previous work evaluated heart rate agreement and validity over the course of a two-mile (3.2 km) trail run (average duration was approximately 22 min), but reliability was not evaluated^20^. Determining the reliability of wearable devices is an issue that has been raised in several systematic reviews^38–40^, but continues to be understudied, perhaps because of the added time investment needed to measure reliability. Because the Consumer Technology Association (CTA) recommends a minimum of 5 min in duration when validating heart rate devices during exercise^41^, this has likely become the minimum default length of time for many investigators^7,18,42^. The consequences of differing exercise durations on decisions relating to validity and reliability of heart rate-based devices is, to our knowledge, unaddressed.

One difficulty is there are no universally accepted standards utilized for the processing of heart rate data. Various organizations have set forth recommendations^41,43^, but as evidenced by the variety of approaches highlighted above, investigators have yet to put these guidelines into practice. In 2018, the CTA published a report recommending that data processing be accomplished through the temporal averaging of the experimental and criterion devices and synced according to the sampling rate of the experimental device^41^. More recently, in 2021 a group of European universities started an initiative to develop and recommend best practices for validating heart rate measurements by consumer wearables (Towards Intelligent Health and Well-Being: Network of Physical Activity Assessment, or INTERLIVE)^43^. Like the CTA, the group recommended that the criterion measure be aligned with the experimental epoch. The group went a step further by recommending that the average measurement window be 5 s or fewer and that an automated synchronization process be implemented^43^.

To date, an unanswered question remains regarding what effect heart rate data processing has on decisions made with respect to wearable technology device agreement, equivalence^44^, reliability, and validity. It is hypothesized that data processing will affect whether wearable technology devices are considered valid and reliable according to predetermined thresholds. Additionally, there is a need to evaluate the effect of a minimal duration versus an entire exercise bout when performed in an outdoor setting. In this regard, we hypothesize that exercise duration should not affect decisions when heart rate is measured concurrently. Finally, as the experimental wearable devices utilized in the current investigation have not been determined to be valid or reliable in any use case, there is a need for this information to be reported. Toward this end, the three main purposes of the study were to (1) determine the effect of heart rate data processing on metrics used to make decisions regarding validity and reliability, (2) evaluate the effect of differing lengths of sampling duration on measures associated with heart rate validity, agreement, equivalence, and reliability, and (3) report the concurrent heart rate validity and reliability of the Polar Verity Sense and Polar OH1 during a trail running use case.

## Results

### Validity

When the entire duration of the trail run was considered, the Polar Verity Sense met the minimum threshold for validity under all data processing methods (see Table 1, Bland–Altman plots are provided in the Supplementary file Figs. S1–S7). When only the first 5 min of the trail run were considered, the Polar Verity Sense did not meet either of the predetermined validity thresholds for any of the data processing methods (see Table 2, Bland–Altman plots are provided in the Supplementary file Figs. S8–S14).

**Table 1:** Polar Verity Sense, entire trail run.

Criterion	Sec-sec	CS 15 s	CS 30 s	CS 1 min	AVG 5 s	AVG 10 s	AVG session
Polar H10	162.5 (26.7)	162.1 (27.2)	161.8 (27.5)	160.8 (28.6)	162.5 (26.7)	162.5 (26.7)	162.9 (12.5)

Validity	Datapoints	Heart rate (bpm)	MAE (bpm)	MAPE (%)	Bias	Limits of agreement	CCC
Verity sec-sec	38,134	159.9 (28.5)	4.2	2.7	2.6	23.6 to − 18.4	0.92
Verity CS 15 s	2554	159.5 (28.8)	4.2	2.7	2.7	24.0 to − 18.7	0.92
Verity CS 30 s	1328	159.2 (28.9)	4.1	2.7	2.6	22.7 to − 17.6	0.93
Verity CS 1 min	640	158.6 (29.4)	4.0	2.6	2.2	21.3 to − 16.9	0.94
Verity AVG 5 s	7618	159.9 (28.4)	4.2	2.7	2.6	23.3 to − 18.0	0.92
Verity AVG 10 s	3804	159.9 (28.4)	4.0	2.6	2.6	23.1 to − 17.8	0.92
Verity AVG session	30	160.2 (11.5)	2.7	1.6	2.7	9.9 to − 4.5	0.93

Equivalence	Datapoints	TOST lower *p*	TOST upper *p*	Lower estimate	Upper estimate	Lower 90% CI	Upper 90% CI
Verity sec-sec	38,134	1.000	< 0.001	− 2.62	2.62	2.29	2.94
Verity CS 15 s	2554	1.000	< 0.001	− 2.68	2.68	1.39	3.97
Verity CS 30 s	1328	1.000	< 0.001	− 2.64	2.64	0.84	4.44
Verity CS 1 min	640	0.871	< 0.001	− 2.30	2.30	− 0.33	4.93
Verity AVG 5 s	7618	1.000	< 0.001	− 2.63	2.63	1.89	3.36
Verity AVG 10 s	3804	1.000	< 0.001	− 2.65	2.65	1.61	3.69
Verity AVG session	30	0.760	0.154	− 2.76	2.76	− 2.54	8.06

Reliability	Datapoints	Heart rate 1 (bpm)	Heart rate 2 (bpm)	CV (%)	ICC		
Verity sec-sec	19,067	159.8 (28.4)	160.0 (28.5)	1.2	0.96		
Verity CS 15 s	1277	159.4 (28.8)	159.6 (28.9)	1.1	0.96		
Verity CS 30 s	664	159.1 (28.9)	159.3 (29.0)	1.0	0.98		
Verity CS 1 min	320	158.6 (29.3)	158.6 (29.6)	0.9	0.98		
Verity AVG 5 s	3809	159.8 (28.3)	160.0 (28.5)	1.2	0.97		
Verity AVG 10 s	1902	159.8 (28.3)	160.0 (28.5)	1.1	0.97		
Verity AVG session	17	160.7 (11.3)	160.7 (10.9)	0.5	0.99		

*sec-sec* second-by-second, *CS* cross sectional, *AVG* average, *bpm* beats per minute, *MAPE* mean absolute percent error, *CCC* Lin’s Concordance Correlation Coefficient, *TOST* two one-sided tested, *CV* coefficient of variation, *ICC*  intraclass correlation coefficient, *CI* confidence interval.

**Table 2:** Polar Verity Sense, first 5-mintues of the trail run.

Criterion	Sec-sec	CS 15 s	CS 30 s	CS 1 min	AVG 5 s	AVG 10 s	AVG session
Polar H10	138.2 (38.8)	136.4 (39.1)	133.7 (39.1)	130.4 (39.3)	138.2 (38.8)	138.2 (38.8)	138.2 (21.6)

Validity	Datapoints	Heart rate (bpm)	MAE (bpm)	MAPE (%)	Bias	Limits of agreement	CCC
Verity sec-sec	9000	128.6 (37.1)	11.0	7.6	9.7	45.3 to − 25.9	0.86
Verity CS 15 s	600	127.0 (36.6)	10.7	7.3	9.4	44.0 to − 25.2	0.87
Verity CS 30 s	300	124.2 (35.8)	10.5	7.2	9.5	43.3 to − 24.3	0.87
Verity CS 1 min	150	121.9 (35.3)	9.9	7.1	8.8	41.6 to − 24.0	0.88
Verity AVG 5 s	1800	128.6 (37.0)	10.9	7.5	9.7	44.8 to − 25.4	0.86
Verity AVG 10 s	900	128.6 (36.9)	10.6	7.2	9.7	44.4 to − 25.0	0.86
Verity AVG session	30	128.6 (19.2)	9.7	6.5	9.7	33.3 to − 13.9	0.74

Equivalence	Datapoints	TOST lower *p*	TOST upper *p*	Lower estimate	Upper estimate	Lower 90% CI	Upper 90% CI
Verity sec-sec	9000	1.000	< 0.001	− 9.7	9.7	8.8	10.6
Verity CS 15 s	600	1.000	< 0.001	− 9.5	9.5	5.9	13.1
Verity CS 30 s	300	0.999	< 0.001	− 9.7	9.7	4.6	14.7
Verity CS 1 min	150	0.976	0.014	− 9.1	9.1	1.90	16.2
Verity AVG 5 s	1800	1.000	< 0.001	− 9.7	9.7	7.6	11.8
Verity AVG 10 s	900	1.000	< 0.001	− 9.8	9.8	6.8	12.7
Verity AVG session	30	0.981	0.012	− 11.3	11.3	2.8	19.8

Reliability	Datapoints	Heart rate 1 (bpm)	Heart rate 2 (bpm)	CV (%)	ICC		
Verity sec-sec	4500	129.0 (37.2)	128.1 (37.0)	3.5	0.94		
Verity CS 15 s	300	127.6 (36.4)	126.4 (36.8)	2.8	0.95		
Verity CS 30 s	150	124.8 (36.3)	123.6 (35.4)	2.5	0.96		
Verity CS 1 min	75	122.5 35.8)	121.4 (35.10	2.2	0.97		
Verity AVG 5 s	900	129.0 (37.1)	128.1 (36.9)	3.3	0.94		
Verity AVG 10 s	450	129.0 (37.0)	128.1 (36.9)	3.3	0.94		
Verity AVG session	15	129.0 (19.8)	128.1 (19.2)	1.7	0.96		

*sec-sec* second-by-second, *CS* cross sectional, *AVG* average, *bpm* beats per minute, *MAPE* mean absolute percent error, *CCC* Lin’s Concordance Correlation Coefficient, *TOST* two one-sided tested, *CV* coefficient of variation, *ICC* intraclass correlation coefficient, *CI* confidence interval.

When the entire duration of the trail run was considered, the Polar OH1 met the minimum mean absolute percent error (MAPE) threshold for validity under all of the data processing methods but did not meet the minimum Lin’s Concordance threshold (see Table 3, Bland–Altman plots are provided in the Supplementary file Figs. S15–S21). When only the first 5 min of the trail run were considered, the Polar OH1 did not meet either of the predetermined validity thresholds for any of the data processing methods (see Table 4, Bland–Altman plots are provided in the Supplementary file Figs. S22–S28).

**Table 3:** Polar OH1, entire trail run.

Criterion	Sec-sec	CS 15 s	CS 30 s	CS 1 min	AVG 5 s	AVG 10 s	AVG Session
Polar H10	162.5 (26.7)	162.1 (27.2)	161.8 (27.5)	160.8 (28.6)	162.5 (26.7)	162.5 (26.7)	162.9 (12.5)

Validity	Datapoints	Heart rate (bpm)	MAE (bpm)	MAPE (%)	Bias	Limits of agreement	CCC
OH1 sec-sec	38,134	157.3 (28.5)	6.8	4.1	5.2	35.2 to − 24.8	0.83
OH1 CS 15 s	2554	156.9 (28.9)	6.8	4.1	5.2	35.2 to − 24.8	0.84
OH1 CS 30 s	1328	156.5 (29.5)	6.8	4.2	5.3	35.6 to − 25.0	0.84
OH1 CS 1 min	640	155.8 (29.6)	6.4	4.0	5.0	33.9 to − 23.9	0.86
OH1 AVG 5 s	7618	157.3 (28.5)	6.7	4.1	5.2	35.0 to − 24.7	0.83
OH1 AVG 10 s	3804	157.3 (28.4)	6.6	4.0	5.2	34.8 to − 24.4	0.84
OH1 AVG session	30	157.3 (13.1)	5.6	3.3	5.6	26.4 to − 15.2	0.60

Equivalence	Datapoints	TOST lower *p*	TOST upper *p*	Lower estimate	Upper estimate	Lower 90% CI	Upper 90% CI
OH1 sec-sec	38,134	1.000	< 0.001	− 5.17	5.17	4.84	5.50
OH1 CS 15 s	2554	0.997	< 0.001	− 5.23	5.23	3.94	6.52
OH1 CS 30 s	1328	0.975	< 0.001	− 5.35	5.35	3.53	7.17
OH1 CS 1 min	640	0.998	< 0.001	− 5.07	5.07	2.40	7.75
OH1 AVG 5 s	7618	1.000	< 0.001	− 5.19	5.19	4.45	5.92
OH1 AVG 10 s	3804	1.000	< 0.001	− 5.21	5.21	4.17	6.25
OH1 AVG session	30	0.935	0.036	− 5.67	5.67	0.054	11.3

Reliability	Datapoints	Heart rate 1 (bpm)	Heart rate 2 (bpm)	CV (%)	ICC		
OH1 sec-sec	19,067	157.6 (29.0)	156.6 (29.0)	3.1	0.85		
OH1 CS 15 s	1277	157.4 (29.4)	156.5 (28.5)	2.9	0.86		
OH1 CS 30 s	664	156.9 (30.2)	156.1 (28.9)	3.1	0.86		
OH1 CS 1 min	320	156.2 (29.9)	155.3 (29.4)	2.6	0.88		
OH1 AVG 5 s	3809	157.8 (28.8)	156.9 (28.2)	2.8	0.86		
OH1 AVG 10 s	1902	157.8 (28.7)	156.9 (28.2)	2.7	0.86		
OH1 AVG session	17	158.4 (11.8)	157.7 (13.5)	2.5	0.57		

*sec-sec* second-by-second, *CS* cross sectional, *AVG* average, *bpm* beats per minute, *MAPE* mean absolute percent error, *CCC* Lin’s Concordance Correlation Coefficient, *TOST* two one-sided tested, *CV* coefficient of variation, *ICC* intraclass correlation coefficient, *CI* confidence interval.

**Table 4:** Polar OH1, first 5 min.

Criterion	Sec-sec	CS 15 s	CS 30 s	CS 1 min	AVG 5 s	AVG 10 s	AVG Session
Polar H10	138.2 (38.8)	136.4 (39.1)	133.7 (39.1)	130.7 (39.3)	138.2 (38.8)	138.2 (38.8)	138.2 (21.6)

Validity	Datapoints	Heart rate (bpm)	MAE (bpm)	MAPE (%)	Bias	Limits of agreement	CCC
OH1 sec-sec	9000	126.1 (35.3)	13.9	9.1	12.2	54.5 to − 30.1	0.79
OH1 CS 15 s	600	124.3 (35.3)	13.8	9.1	12.1	54.6 to − 30.4	0.79
OH1 CS 30 s	300	121.2 (35.4)	13.8	9.3	12.6	55.5 to − 30.4	0.78
OH1 CS 1 min	150	120.1 (33.6)	12.7	8.6	10.6	50.9 to − 29.6	0.81
OH1 AVG 5 s	1800	126.1 (35.2)	13.7	8.9	12.2	54.1 to − 29.7	0.79
OH1 AVG 10 s	900	126.1 (35.1)	13.4	8.7	12.2	53.5 to − 29.1	0.79
OH1 AVG session	30	126.1 (20.3)	12.3	8.3	12.2	42.0 to − 17.6	0.63

Equivalence	Datapoints	TOST lower *p*	TOST upper *p*	Lower estimate	Upper estimate	Lower 90% CI	Upper 90% CI
OH1 sec-sec	9000	1.000	< 0.001	− 12.2	12.2	11.3	13.10
OH1 CS 15 s	600	1.000	< 0.001	− 12.2	12.2	8.6	15.7
OH1 CS 30 s	300	1.000	< 0.001	− 12.7	12.7	7.7	17.7
OH1 CS 1 min	150	0.993	0.004	− 10.9	10.9	3.90	17.9
OH1 AVG 5 s	1800	1.000	< 0.001	− 12.2	12.2	10.2	14.3
OH1 AVG 10 s	900	1.000	< 0.001	− 12.2	12.2	9.4	15.1
OH1 AVG session	30	0.993	0.004	− 13.8	13.8	5.0	22.6

Reliability	Datapoints	Heart rate 1 (bpm)	Heart rate 2 (bpm)	CV (%)	ICC		
OH1 sec-sec	4500	125.8 (35.8)	126.3 (34.8)	4.0	0.92		
OH1 CS 15 s	300	124.0 (36.2)	124.6 (34.6)	4.2	0.92		
OH1 CS 30 s	150	120.7 (36.9)	121.7 (33.8)	5.0	0.90		
OH1 CS 1 min	75	120.1 (33.8)	120.2 (33.5)	3.0	0.95		
OH1 AVG 5 s	900	125.8 (35.7)	126.3 (34.7)	3.9	0.93		
OH1 AVG 10 s	450	125.8 (35.4)	126.3 (34.7)	3.7	0.93		
OH1 AVG session	15	125.8 (21.1)	126.3 (20.1)	2.9	0.94		

*sec-sec* second-by-second, *CS* cross sectional, *AVG* average, *bpm* beats per minute, *MAPE* mean absolute percent error, *CCC* Lin’s Concordance Correlation Coefficient, *TOST* two one-sided tested, *CV* coefficient of variation, *ICC* intraclass correlation coefficient, *CI* confidence interval.

### Equivalence

When the entire duration of the trail run was considered, the Polar Verity Sense did not meet the assumption of equivalence for any of the data processing methods (see Table 1, equivalence plots are provided in the Supplementary file Figs. S29–S35). The device did not meet the assumption when only the first 5 min of the trail run were considered (see Table 2, equivalence plots are provided in the Supplementary file Figs. S36–S42).

Similar to what was observed for the Polar Verity Sense, the OH1 did not meet the assumption of equivalence for any of the data processing methods when the entire trail run was considered, or when only the first 5 min of the run were considered (see Tables 3 and 4, equivalence plots are provided in the Supplementary file Figs. S43–S56).

### Reliability

The Polar Verity Sense met the threshold for both absolute reliability (coefficient of variation, CV) and relative reliability (intraclass correlation coefficient, ICC) for all data processing methods when the entire duration of the trail run was considered (see Table 1). The same observations were noted when only the first 5 min of the trail run were considered (see Table 2).

The Polar OH1 met all thresholds for reliability over the course of the entire trail run except when considering the session average heart rate method (see Table 3). The session average did not meet the assumption for ICC. When only the first 5 min were considered, the Polar OH1 met the threshold for all reliability tests for all of the data processing methods (see Table 4).

### Power and sample size determination

Trail running is an inherently dynamic exercise that produces a variable, rather than steady state, heart rate response. With this acknowledgement, we report the actual power derived from each of the data processing methods along with a calculated sample size (see Table 5). The aim is to provide subsequent researchers with information necessary to determine appropriate sample sizes for similar use cases.

**Table 5:** Actual power and sample size calculations.

	Entire run				First 5-min			
	*r*	*r*^2^	Power	Sample size	*r*	*r*^2^	Power	Sample size
Verity sec-sec	0.9270	0.8593	0.8761	5	0.8864	0.7857	0.8755	6
Verity CS 15 s	0.9260	0.8575	0.8737	5	0.8935	0.7983	0.8886	6
Verity CS 30 s	0.9350	0.8742	0.8956	5	0.8976	0.8057	0.8029	5
Verity CS 1 min	0.9440	0.8911	0.9168	5	0.9073	0.8233	0.8054	5
Verity AVG 5 s	0.9290	0.8630	0.8810	5	0.8897	0.7915	0.8816	6
Verity AVG 10 s	0.9300	0.8649	0.8835	5	0.8915	0.7948	0.8850	6
Verity AVG session	0.9570	0.9158	0.8322	4	0.8322	0.6926	0.8479	7
OH1 sec-sec	0.8480	0.7191	0.8004	6	0.8345	0.6963	0.8516	7
OH1 CS 15 s	0.8530	0.7276	0.8104	6	0.8352	0.6976	0.8530	7
OH1 CS 30 s	0.8550	0.7310	0.8144	6	0.8315	0.6913	0.8466	7
OH1 CS 1 min	0.8730	0.7621	0.8499	6	0.8529	0.7274	0.8102	6
OH1 AVG 5 s	0.8500	0.7225	0.8045	6	0.8371	0.7008	0.8562	7
OH1 AVG 10 s	0.8520	0.7259	0.8084	6	0.8415	0.7081	0.8634	7
OH1 AVG session	0.6570	0.4316	0.8034	12	0.7368	0.5429	0.8045	9

*sec-sec* second-by-second, *CS* cross sectional, *AVG* average, *r* Pearson’s *r*, *r*^*2*^ coefficient of determination.

Considering the Polar Verity Sense over the course of the entire trail run period, the actual power ranged from 0.8575 (15-s cross-sectional sampling) to 0.9158 (average heart rate across the entire session). Power analyses using these data revealed an appropriate total sample size to be four to five participants. When only the first 5 min of the trail run were considered, the actual power ranged from 0.8029 (30-s cross-sectional sampling) to 0.8886 (15-s cross-sectional sampling). Power analyses using these data revealed an appropriate total sample size to be five to seven participants.

When the Polar OH1 was considered over the entire trail run duration, the actual power ranged from 0.8004 (second-by-second cross-sectional sampling) to 0.8499 (1-min cross-sectional sampling). Power analyses using these data revealed an appropriate total sample size to be six to twelve participants. When only the first 5 min of the trail run were considered, the actual power ranged from 0.8045 (session average) to 0.8634 (10-s averages). Power analyses using these data revealed an appropriate total sample size to be six to nine participants.

## Discussion

The three-fold purpose of this investigation was to (1) determine the effect of heart rate data processing methods on assumptions used to make validity and reliability decisions, (2) evaluate the effect of different lengths of sampling duration on measures associated with heart rate validity, agreement, equivalence, and reliability, and (3) report concurrent heart rate validity and reliability of the Polar Verity Sense and Polar OH1 during trail running. Differences in data processing methods did not affect the interpretation of the Polar Verity Sense heart rate data. The same observations were true for the Polar OH1, with the exception of the overall session average, which was not aligned with the remaining data processing methods. Considering the duration of data processing, utilizing only the first 5 min of the trail run affected agreement (increased bias and limits of agreement) and validity (increased MAPE and lower CCC) measurements for both devices but not equivalence or reliability metrics when evaluated against the entire duration of the run. Overall, these findings provide evidence that the Polar Verity Sense is both valid and reliable for heart rate measurements during a trail running use case. The utility of the Polar OH1 depends on how the heart rate data are processed.

To determine if utilizing different data processing methods would affect decisions related to the reliability and validity of the experimental wearable technology devices, a variety of methods were employed in the current study. The methods have been commonly used in the literature, and include a cross-sectional approach, evaluating a single measurement second-by-second^7,15–23^, every 15 s^24^, 30 s^25^, and 60 s^25–30^. We also evaluated the effect of smoothing heart rate data by taking an average over time, including 5-s epochs^31–34^, 10-s epochs^35^, and an average of the entire session^37^ as have been reported in the literature. Our findings reveal that the Polar Verity Sense was considered both reliable and valid over the duration of the entire trail run regardless of the data processing method used. Our findings of the Polar OH1 are mixed, with the average of the entire session not meeting the predetermined threshold for reliability (specifically the ICC). Additionally, the Polar OH1 did not meet the validity threshold for CCC using any of the data processing methods. It should be noted that the average of the entire session contained the least number of data points (17 versus 320 to 19,067 for the other methods), although evidence exists to suggest that an appropriate number of participants were tested and sufficient power was obtained. It is tempting to speculate that a small number of data points may not affect decisions on wearable devices that should be considered reliable and valid but may expose devices where the assumptions cannot be met. Further investigation into the consequences of these findings is warranted.

The Consumer Technology Association recommends a minimum duration of 5 min when validating heart rate devices during an exercise use case^41^. Because of this recommendation, 5 min may be the preferred length of time used for validation studies^7,18,42^. Since we previously recommended utilizing longer time periods in applied settings^20^, we wanted to determine what effect evaluating only the first 5 min of the trail run would have on common assumptions, contrasting them with the entire duration of the session. The Polar Verity Sense met the minimum thresholds for MAPE and CCC when the entire run was considered but neither threshold when only the first 5 min were considered. This case is peculiar, as concurrent device validity should theoretically be expected to meet the predetermined thresholds regardless of the duration employed (i.e. a valid heart rate device will report accurate measures regardless of terrain inclines or how variable the heart rate response is to exercise). These data raise questions of interest that warrants further investigation. The first question is associated with the quantity of data reported—namely, whether more data consequentially reduces the influence of spurious readings from a device. Evidence from the current investigation suggests this may be the case, particularly the interpretation of the Polar OH1 data over the entire run when considering the session average against all other data processing methods. Another question centers on the frequency of such spurious readings, and whether they are more likely to occur at the outset of an exercise bout before a steady state is reached. While this potential explanation is intriguing, we previously reported no change in heart rate assumptions during the uphill portion (initial portion of a trail run) when compared to the downhill portion of a trail run (latter portion)^20^. It is clear that while much research has focused on the concurrent validity of wearables during exercise^15,18,31,36,45–47^, a greater focus needs to be directed toward the consequences of varying duration and what effect this factor has on ultimate decisions related to device validity and reliability. Additionally, how exercise intensity is varied is important to future investigations. While trail running is an applied activity that is inherently variable, future studies employing consistent variations in intensity (such as high-intensity interval training) are warranted. Furthermore, conducting the same analyses in a wider array of steady state aerobic exercises (such as cycling, swimming, and running), and high-intensity anaerobic exercise would be useful to confirm whether those results are similar to the trail running use case in the current investigation.

The validity of the Polar OH1 has been reported for various use cases including treadmill and cycle exercise^19,23^, swimming^21^, and a variety of training modalities (biking, tennis, running, soccer, walking)^35^. With second-by-second data processing, the Polar OH1 was deemed to have acceptable validity during treadmill (MAPE between 0.2 and 1.9%) and cycle exercise (MAPE between 0.6 and 3.9%)^23^. Employing second-by-second data processing, the Polar OH1 was reported to have acceptable agreement during treadmill and spin bike activities (mean bias less than 1 bpm)^19^. Also utilizing second-by-second processing, the Polar OH1 was deemed to have acceptable validity through all ranges of front crawl swimming intensity (ICC between 0.72 and 0.96)^21^. Using 10-s smoothing, the Polar OH1 was considered to have good agreement, particularly for endurance sports (difference from criterion < 5%), as well as acceptable reliability (ICC = 0.99) although the protocol for determining reliability was not disclosed^35^. We add to the literature that the Polar OH1 may be considered both valid and reliable during trail runs longer than 5 min, with the exception of when the data processing is averaged over the course of the session.

The use of the Polar Verity Sense has been reported in a variety of applications, including during a 24-h ultramarathon^48^, obtaining physiological stress measures in patients on a workplace stress reduction program^49^, and in a proposal to monitor intensity adherence of a frame running program in children with cerebral palsy^50^. To our knowledge, the only published literature on the validity of the Polar Verity Sense is in abstract form from our laboratory group^51–53^, and the reliability of the device has not been established. We report for the first time that the Polar Verity Sense can be considered both valid and reliable during trail runs longer than 5 min.

This investigation is not without limitations. Our previous work has detailed how conducting research in applied settings with ambient light sources could affect wearable devices that rely on photoplethysmography (PPG)^20^. As the present investigation was conducted in an outdoor trail setting, ambient light must be considered a potential limiting factor. Another limitation could lie in the manner in which we evaluated concurrent reliability, utilizing two of the same devices attached to each arm. While this approach has been used with footpod-based devices^54^, the utility has not been employed in PPG-based wearables. Thus, it is possible that differences in blood flow patterns between limbs could have affected reliability measures, making the devices appear unreliable when they were actually reliable. Another limitation is potentially found in the statistical measures used to determine the acceptability of the devices. While no common set of statistical tests are utilized to provide evidence of device acceptability, testing for equivalence has been proposed^44^. A common test of equivalence is the two one-sided test (TOST); unfortunately, appropriate TOST thresholds have not been established for wearable devices^45^. Given the data presented in the current investigation, the utility of the TOST for the determination of acceptability of wearable devices in an applied setting may be limited. This conclusion stems from the observation that equivalence was unacceptable regardless of whether the thresholds for reliability and validity were met. Further investigation into the appropriate use cases of the TOST test in wearable device evaluation are warranted. Finally, a potential limitation could be that we did not test at least twenty participants, as recommended by the CTA^41^. In this regard, we have reported the actual power obtained from each of the data processing methods (Table 5) and provide evidence to suggest that an appropriate number of data points were obtained from enough participants.

The current investigation provides evidence that despite the numerous methods in which wearable device heart rate data are processed, the approach may have little effect on the interpretation of overall validity and reliability, provided an adequate number of data points are obtained from enough participants. If a device is truly valid and reliable, it will meet the minimum thresholds regardless of the number of observations obtained. On the other hand, it is possible that obtaining a large number of observations, such as through second-by-second processing, may artificially inflate the validity or reliability metrics by concealing spurious observations. Considering this possibility, it may be prudent for researchers to perform data processing with both a minimal number of data points (session average) and many data points (i.e., any of the other methods used in this investigation) to tease out their potential effects upon which decisions are made about reliability and validity. The data additionally seem to suggest that, for exercises of highly variable intensity such as trail running, durations longer than 5 min are warranted. With the evidence presented in this study, we conclude that the Polar Verity Sense is both valid and reliable during trail running.

## Methods

### Participants

Seventeen healthy participants (Female *n* = 7; Male *n* = 10; Transgender, Intersex, or Other *n* = 0) completed testing. Demographic characteristics: Age = 25 ± 9 years (mean ± standard deviation), height = 168 ± 9 cm, mass = 72 ± 14 kg. Participants were screened and deemed not to require medical clearance to complete exercise according to the American College of Sports Medicine preparticipation health screening recommendations^55^. Participants were deemed healthy if they had no cardiovascular, metabolic, or renal disease, and had no signs or symptoms suggestive of the diseases. Participants were excluded if they had known cardiovascular, metabolic, or renal disease or if they did not participate in regular exercise and had signs or symptoms associated with the diseases. A power analysis was conducted using our pilot data with the same wearable devices^52^, indicating the need for at least eleven participants (coefficient of determination r^2^ = 0.57, correlation ρ effect size = 0.755, α = 0.05, β = 0.80)^56^. Prior to participation, individuals gave verbal consent and completed an approved informed consent document. The methods were performed in accordance with relevant guidelines and regulations and approved by Southern Utah University (#11-082022a) and the University of Nevada, Las Vegas (UNLV-2022-392).

### Protocol

Participants were outfitted with heart rate sensing wearable devices and a secure Bluetooth connection was confirmed. In all instances, devices were affixed according to manufacturer recommendations. The criterion device was the Polar H10 (Polar Electro, Kempele, Finland) attached securely around the chest of the participant. The experimental devices were the Polar OH1 (Polar Electro, Kempele, Finland) and Polar Verity Sense (Polar Electro, Kempele, Finland), placed on both the right and left biceps. Two of the same models were used simultaneously so that concurrent reliability could be obtained^54^. All devices (H10, Verity Sense, OH1) were connected via Bluetooth to an iPad mini (Apple Inc., Cupertino, CA) with the PerformTek application (Valencell, Inc., Raleigh, NC) which provides second-by-second heart rate of all connected devices on a single csv file.

**Figure 1:**
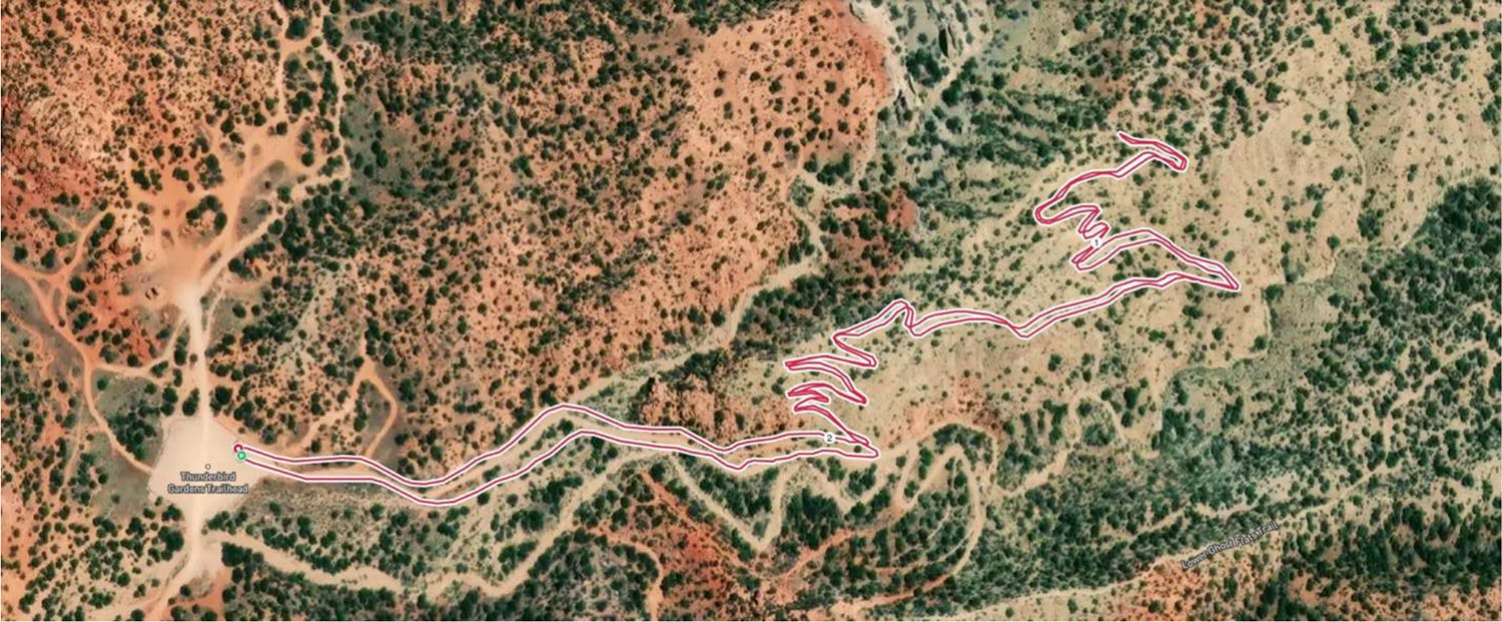
Physical map of the Thunderbird Gardens Lightning Switch trail, where participants (*N* = 17) completed a self-paced 10-min run out, and then returned to the trailhead while connected to the Polar H10 (criterion), and experimental devices (Polar Verity Sense and Polar OH1). The map is a representative training session downloaded by the authors from https://flow.polar.com using the satellite map option.

Participants were instructed to complete a self-paced, out-and-back run on the Thunderbird Gardens Lightning Switch trail in Cedar City, UT (see Fig. 1). Participants ran out on the trail for 10 min in a generally uphill direction and then returned to the trailhead. The mean running time was 21.2 ± 1.6 min (range = 19.5 to 24.3 min). Estimated maximal heart rate was calculated using 211 – (0.64 × age) which formula is accurate for active individuals^57^. Using the highest heart rate obtained from the criterion device during the trail run as a percentage of the age estimated maximal heart rate revealed the exercise bout to be of high intensity (mean = 94.5 ± 4.9%; range = 83.5 to 100.0%). The environmental conditions during testing included the following averages and ranges: temperature = 19.8 ± 4.5 °C (8.9 to 25 °C), humidity = 48.6 ± 20.6% (12 to 86%), windspeed = 14.3 ± 12.4 km h^−1^ (0 to 33.8 km h^−1^). The altitude was 1783 m at the trailhead, and the elevation change was 52.5 ± 11.1 m (36.6 to 72.8 m).

### Devices

#### Polar H10

The Polar H10 chest strap has been shown to be valid compared to electrocardiography^58^, and have acceptable reliability^59^, although the use case specific to trail running has not been determined. The Polar H10 is an electrocardiogram-based heart rate sensor that was secured around the chest of the participant at the level of the xyphoid process. The device contains plastic electrodes on the underside of the strap that detect heart rate. The sensor materials include acrylonitrile butadiene styrene (ABS), ABS plus glass fiber (ABS + GF), polycarbonate, and stainless steel, while the strap material is composed of 38% polyamide, 29% polyurethane, 20% elastane, 13% polyester, and silicone prints. The Polar H10 has a sampling frequency of 1000 Hz. It was connected to an iPad mini via Bluetooth.

#### Polar Verity Sense

The Polar Verity Sense is a PPG device. It is an optical heart rate sensor designed to be worn on the upper arm. The sensor materials include ABS, ABS + GF, poly(methyl methacrylate) (PMMA), and steel use stainless (SUS) 316. The device was positioned with the sensor on the underside of the armband and firmly against the skin. The Polar Verity Sense has a sample rate of 135 Hz and was connected to an iPad mini via Bluetooth.

#### Polar OH1

The Polar OH1 is a PPG device. Like the Polar Verity Sense, it is an optical heart rate sensor designed to be worn on the upper arm. The sensor materials include ABS, ABS + GF, PMMA, and SUS 316. The device was positioned so that the sensor was on the underside of the armband and firmly against the skin. The Polar OH1 has a sample rate of 135 Hz. It was connected to an iPad mini via Bluetooth.

### Data processing

There was no missing data from either of the experimental wearable technology devices or from the criterion device. Data were processed per methods commonly reported in the literature using cross-sectional (CS) and smoothing (or averaging, [AVG]) methods. For the CS approach, data were obtained at each timepoint noted. For the second-by-second method, data were obtained each second (60 times on the second over the course of 60 s). For the 15-s cross-sectional method, data were obtained every 15 s (four times per minute: at 15 s, 30 s, 45 s, and 60 s). For the 30-s cross-sectional method, data were obtained every 30 s (two times per minute: at 30 s and 60 s). For the 60-s cross-sectional method, data were obtained every minute for the duration of the exercise period.

For the AVG approach, data were averaged across the particular timeframe. For the 5-s average method, the mean of the data was obtained in 5-s increments (12 times per minute: 0–5 s, 5–10 s, 10–15 s, 15–20 s, 20–25 s, 25–30 s, 30–35 s, 35–40 s, 40–45 s, 45–50 s, 50–55 s, 55–60 s). For the 10-s average method, the mean of the data was obtained in 10-s increments (six times per minute: 0–10 s, 10–20 s, 20–30 s, 30–40 s, 40–50 s, 50–60 s). For the 30-s average method, the mean of the data was obtained in 30-s increments (two times per minute: 0–30 s and 30–60 s). For the session average, the mean of the entire data set for each participant was utilized (one value per participant).

### Statistical analysis

Measures associated with validity that we reported included mean absolute percent error, and Lin’s Concordance Correlation Coefficient, and the mean absolute error. The equations for these metrics were input into an Excel spreadsheet (Microsoft Excel for Mac version 16.66.1, Redmond, WA). For validity thresholds we have used a MAPE value ≤ 5%^7,20^, and a CCC ≥ 0.90^20^.

Agreement was determined using the Bland–Altman analysis. Bland–Altman bias and limits of agreement were determined using the blandr analysis in jamovi (version 2.3.19.0)^60^. There are currently no thresholds established to denote acceptable agreement on the basis of the Bland–Altman analysis independent of other measures.

Equivalence was determined using the two one-sided test. Equivalence testing was determined using the TOSTER analysis in jamovi (version 2.3.19.0)^60^. If the confidence interval (CI) lies within the upper and lower estimate, the two means are considered equivalent^61^.

Measures associated with reliability that we reported included the coefficient of variation, and intraclass correlation coefficient. The equation for CV was input into an Excel spreadsheet (Microsoft Excel for Mac version 16.66.1, Redmond, WA). Both the ICC and Cronbach’s α were determined using SPSS Statistics (IBM SPSS Statistics, version 28.0.1.0, Chicago, IL). For the outdoor trail setting we used a threshold of ≤ 10% for CV, and ≥ 0.70 for ICC^62^.

SPSS Statistics (IBM SPSS Statistics, version 28.0.1.0, Chicago, IL) were used to determine Pearson’s Product Moment Correlation Coefficients. The r^2^ value was then used in G Power^56^ to determine actual power and sample sizes.

## Supplementary Information

Supplementary Figures.

## Data Availability

The raw dataset generated during the current study are available in the Harvard Dataverse repository, 10.7910/DVN/0M49BY.
