# Supplementary material for: Heart rate processing algorithms and exercise duration on reliability and validity decisions in biceps-worn Polar Verity Sense and OH1 wearables
Source: Sci Rep. 2023 Jul 20;13:11736. doi: 10.1038/s41598-023-38329-w (PMC10359261; doi:10.1038/s41598-023-38329-w)
Supplement: Supplementary file 1 — Supplementary Figures. [file 41598_2023_38329_MOESM1_ESM.pdf]

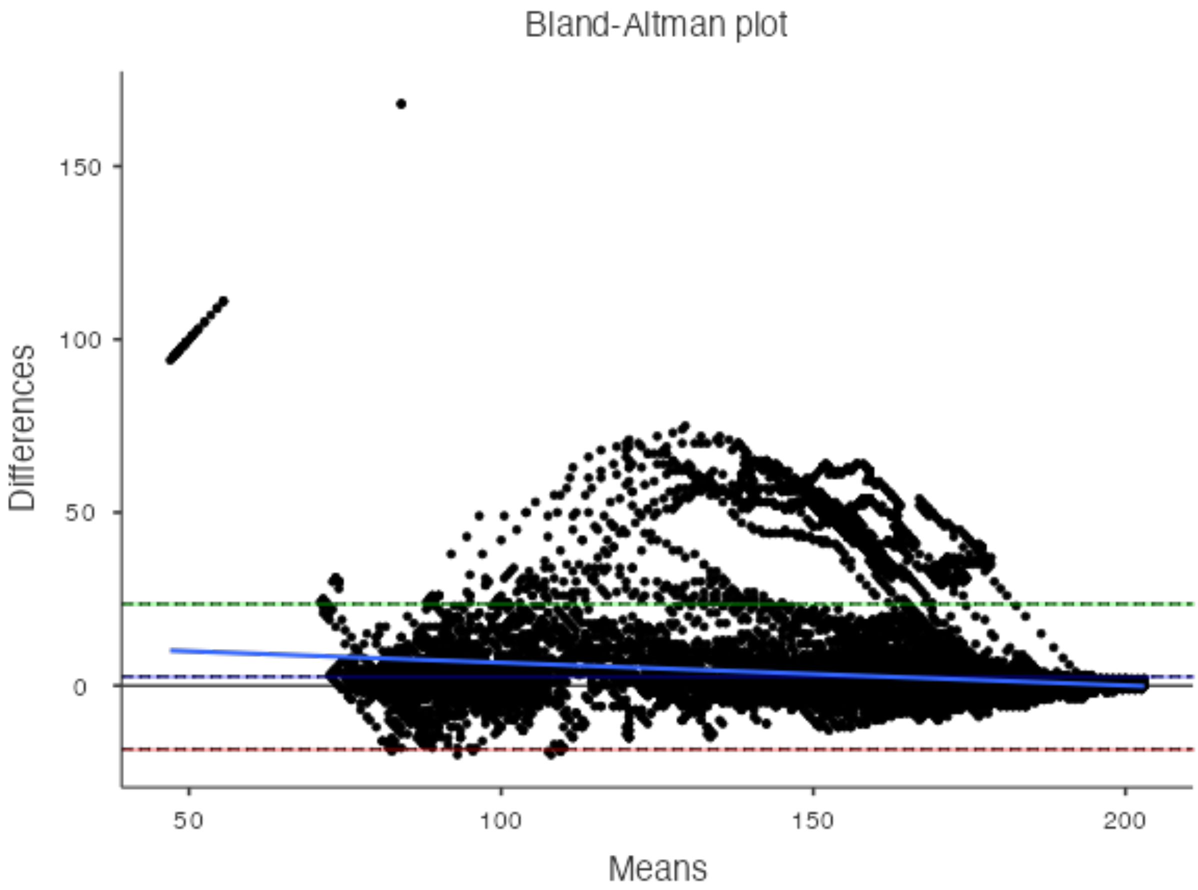

Supplementary Figure S1. Polar Verity Sense second-by-second (entire trail run).

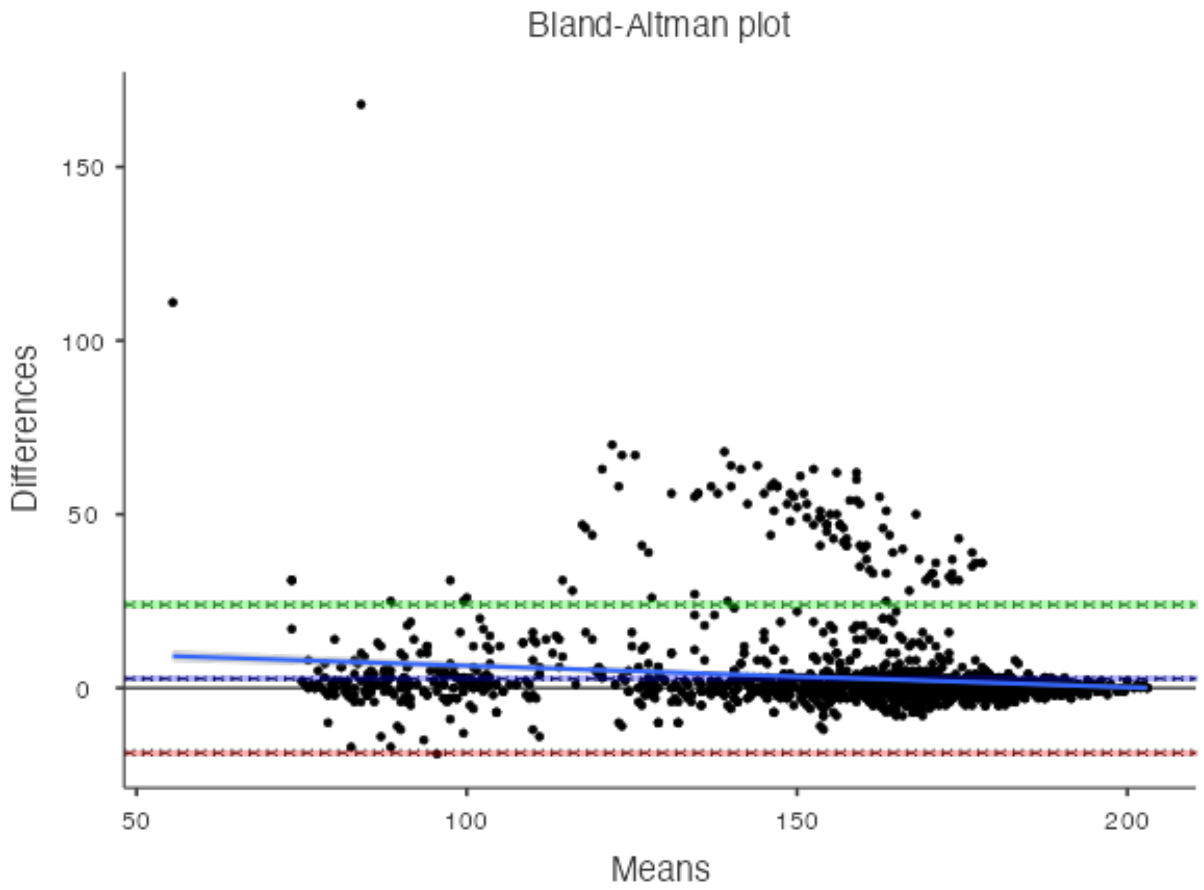

Supplementary Figure S2. Polar Verity Sense 15 second cross-sectional (entire trail run)

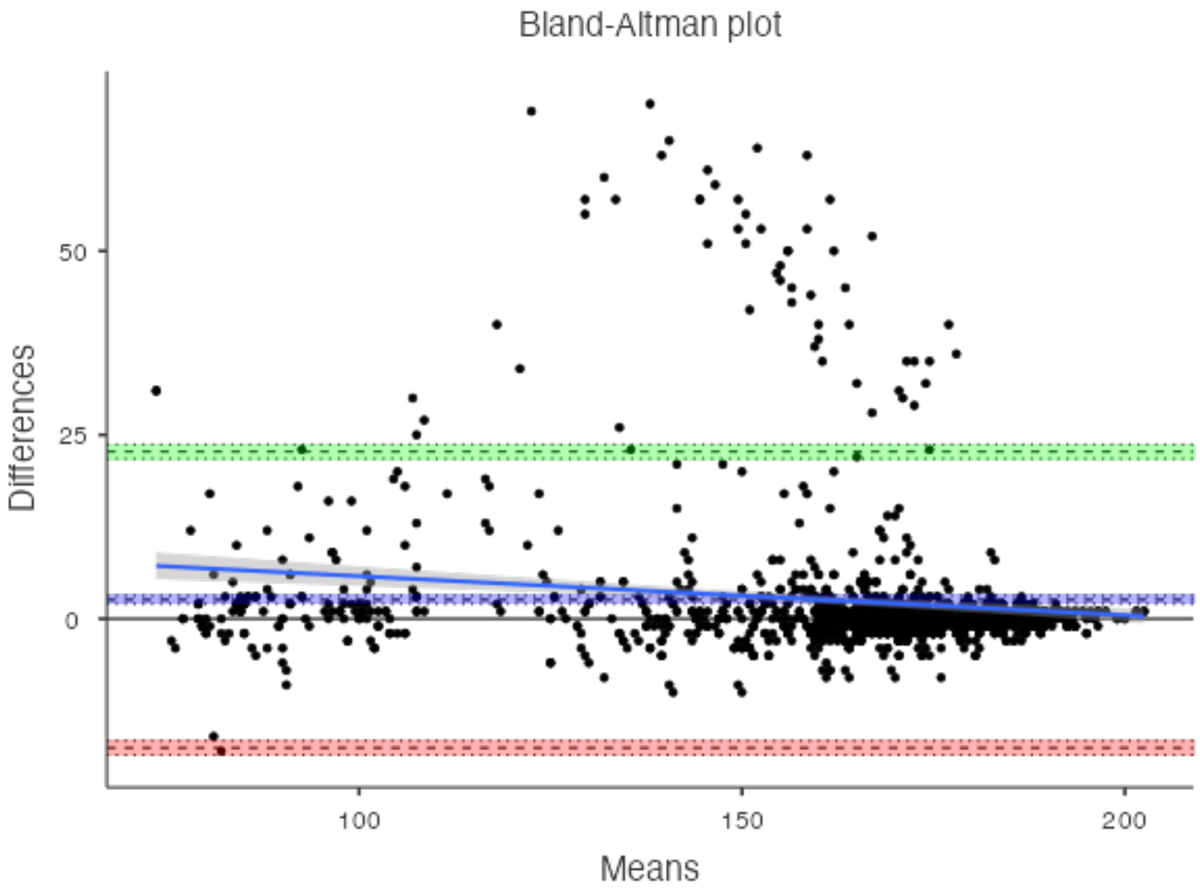

Supplementary Figure S3. Polar Verity Sense 30 second cross-sectional (entire trail run)

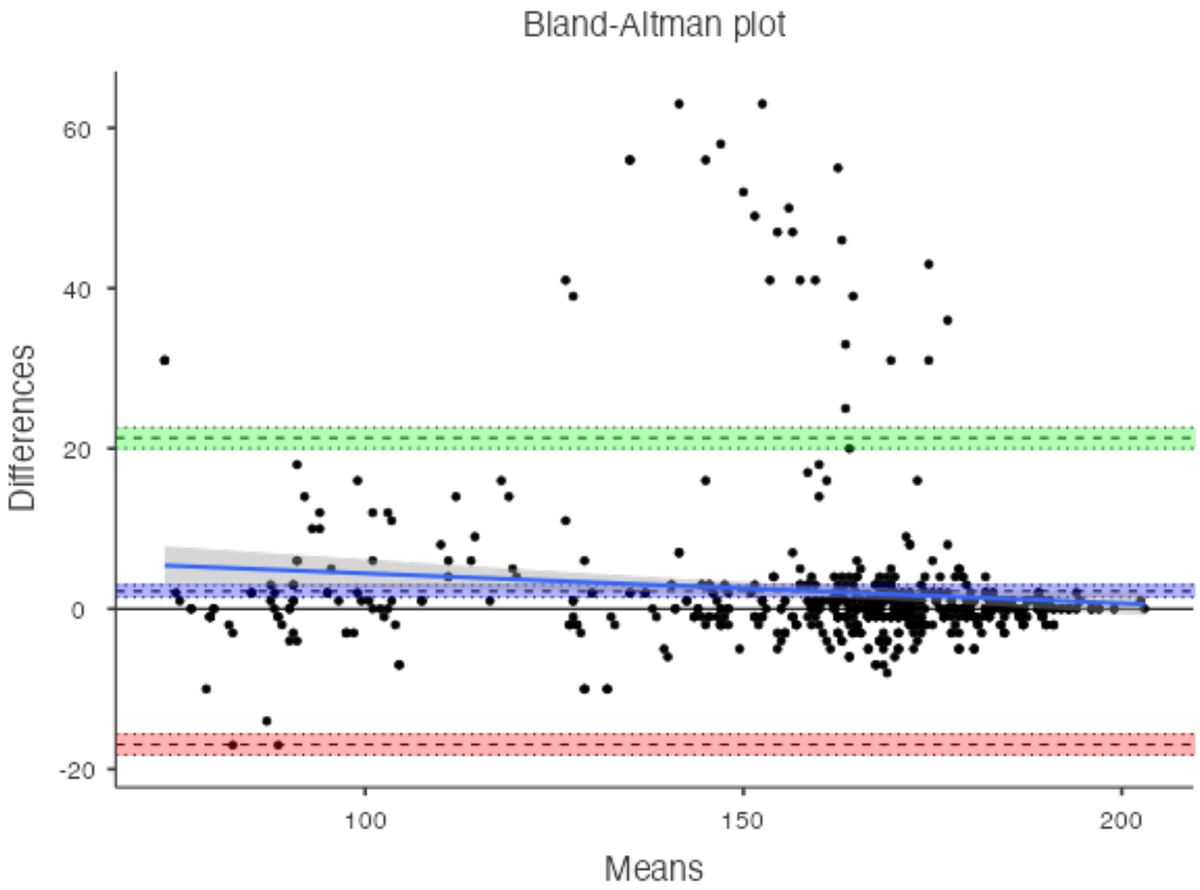

Supplementary Figure S4. Polar Verity Sense 1 minute cross-sectional (entire trail run)

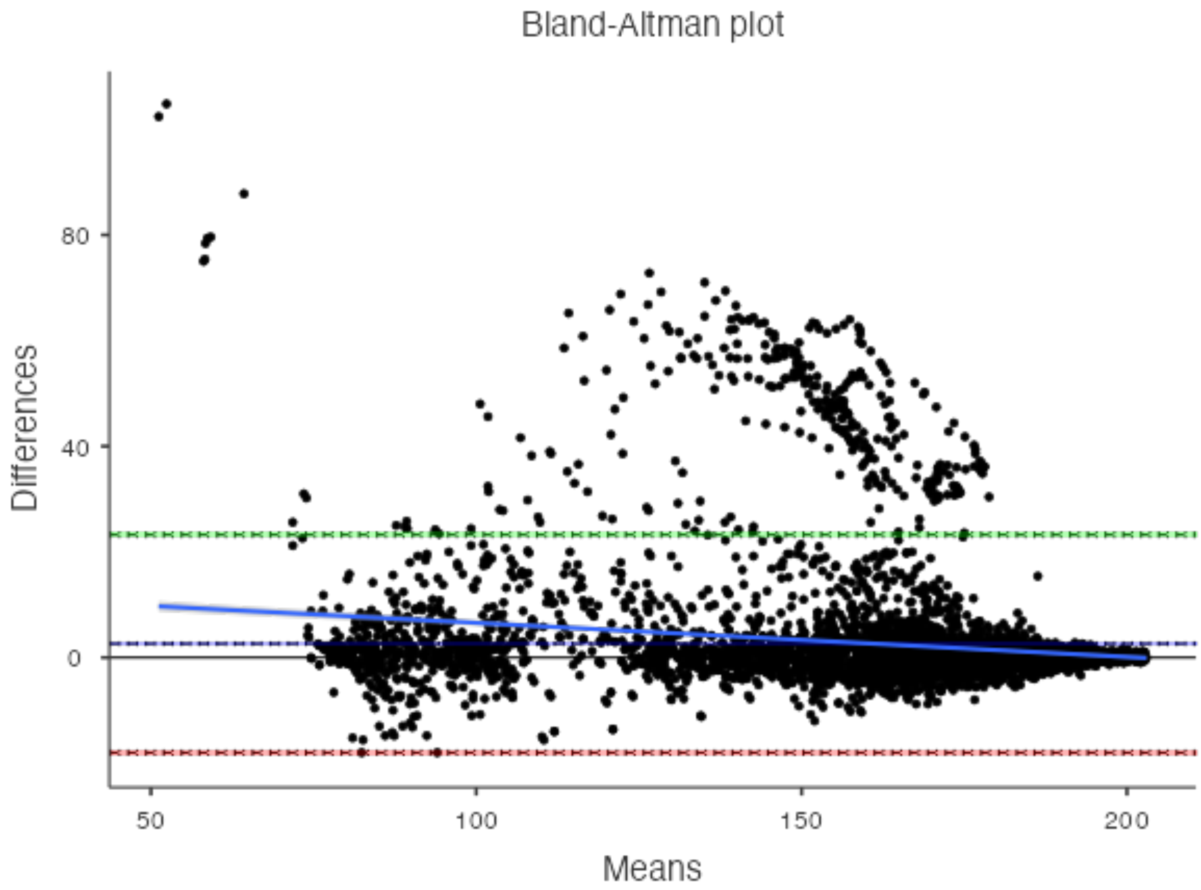

Supplementary Figure S5. Polar Verity Sense 5 second average (entire trail run)

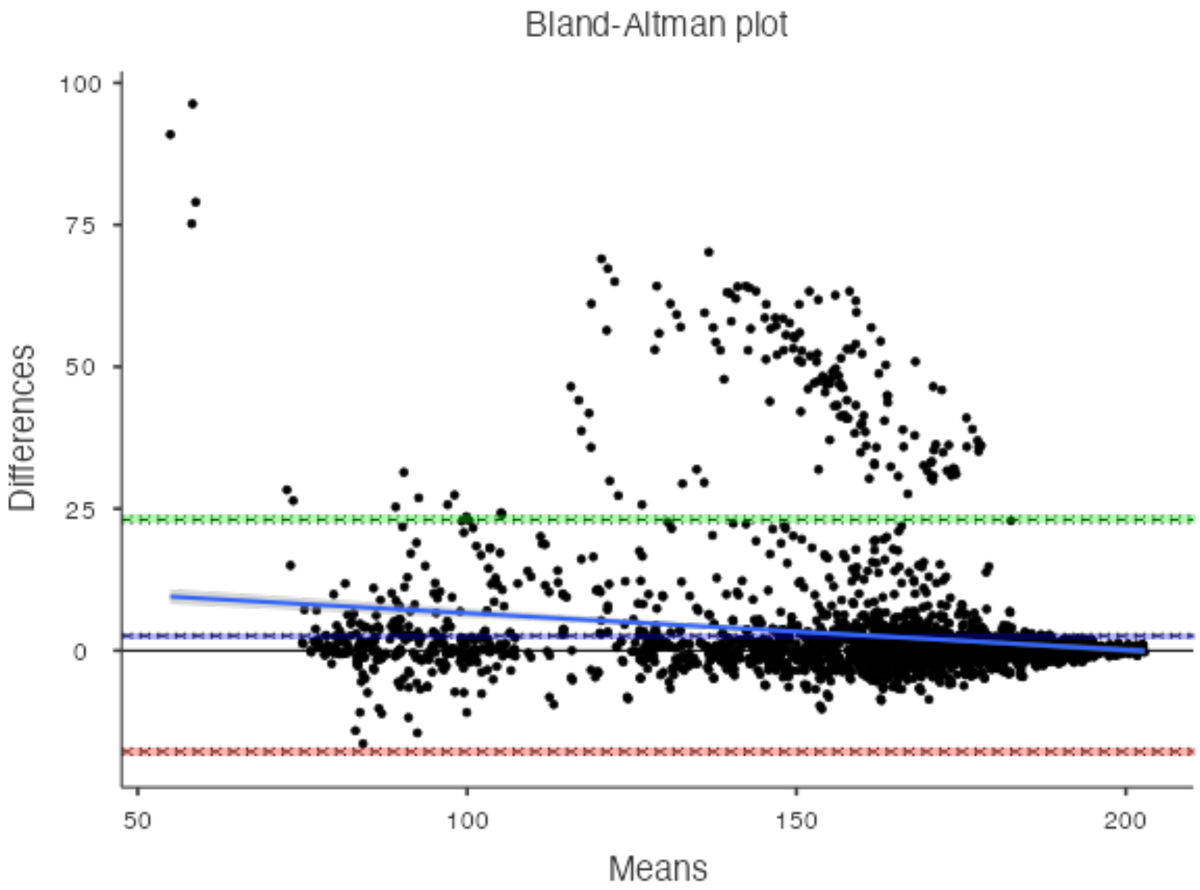

Supplementary Figure S6. Polar Verity Sense 10 second average (entire trail run)

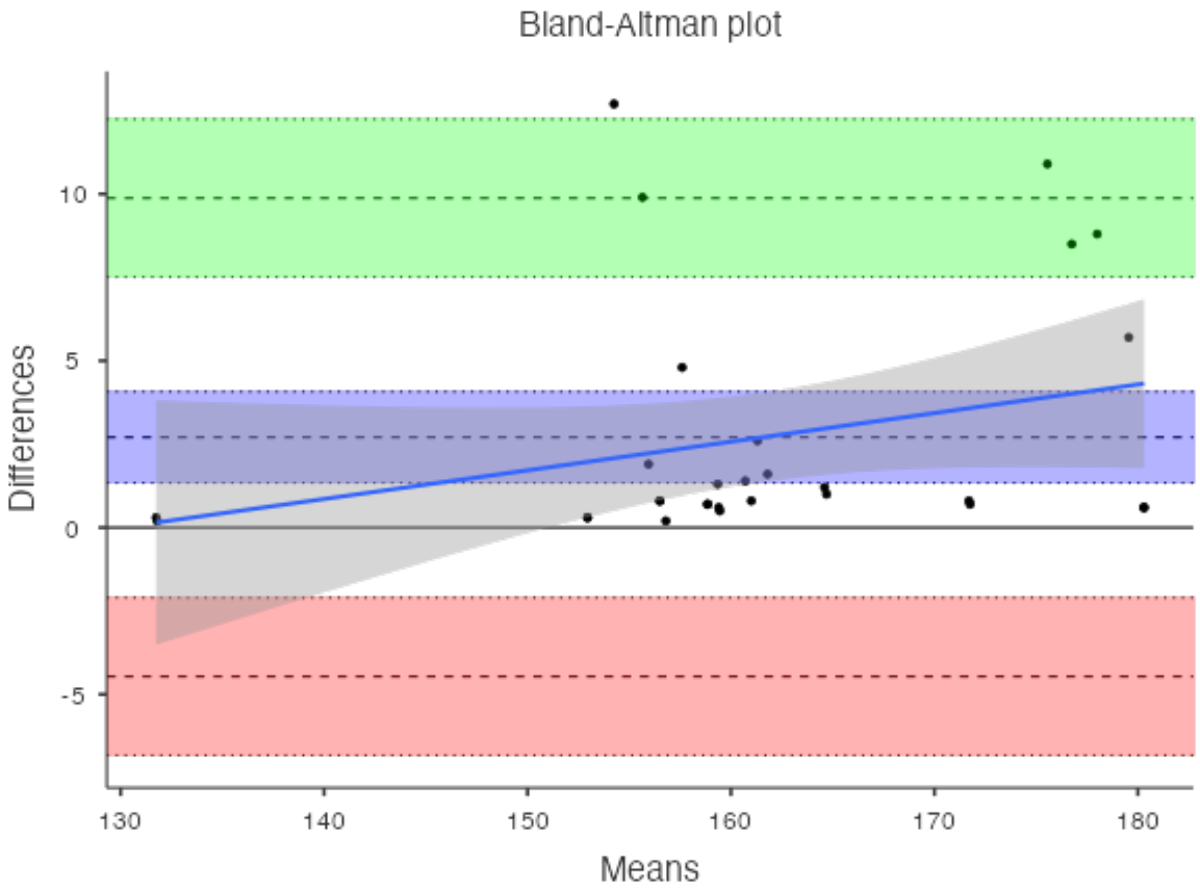

Supplementary Figure S7. Polar Verity Sense session average (entire trail run)

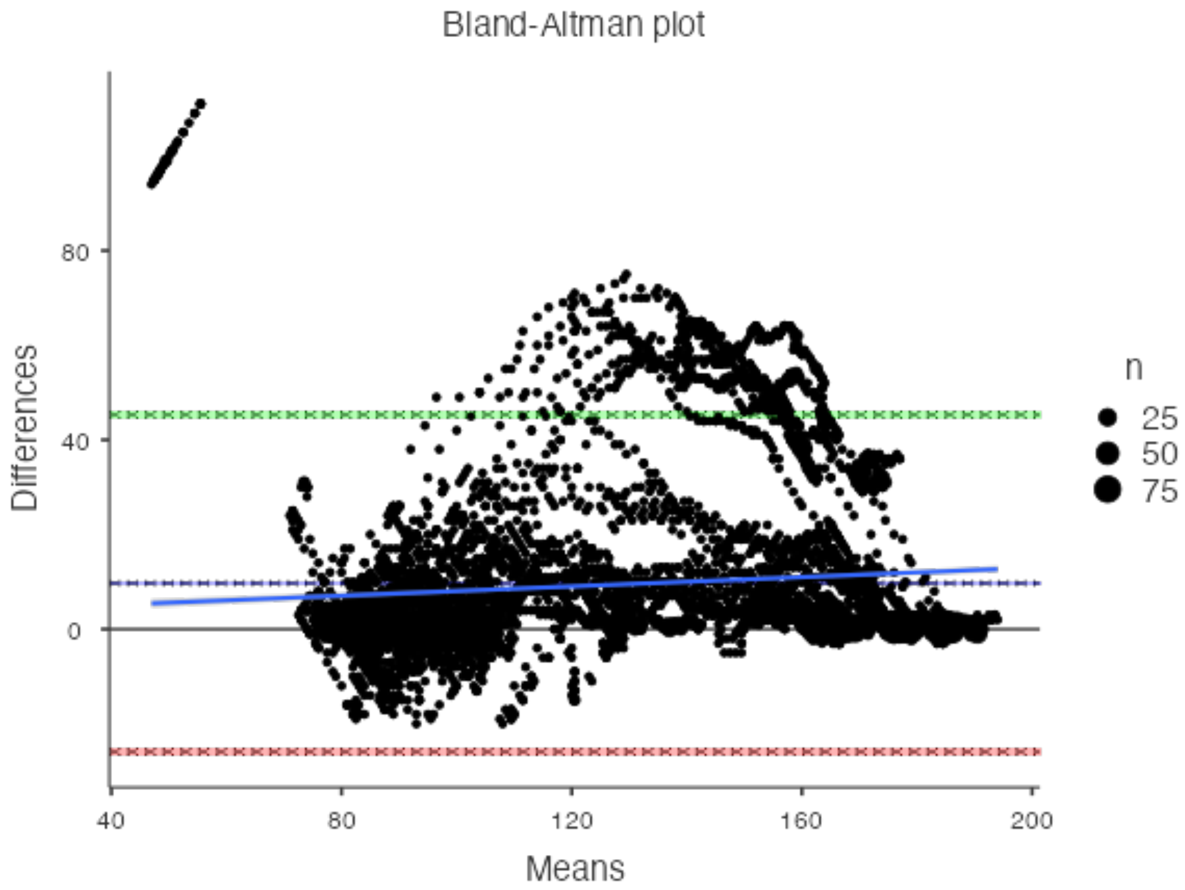

Supplementary Figure S8. Polar Verity Sense second-by-second (first 5 minutes only)

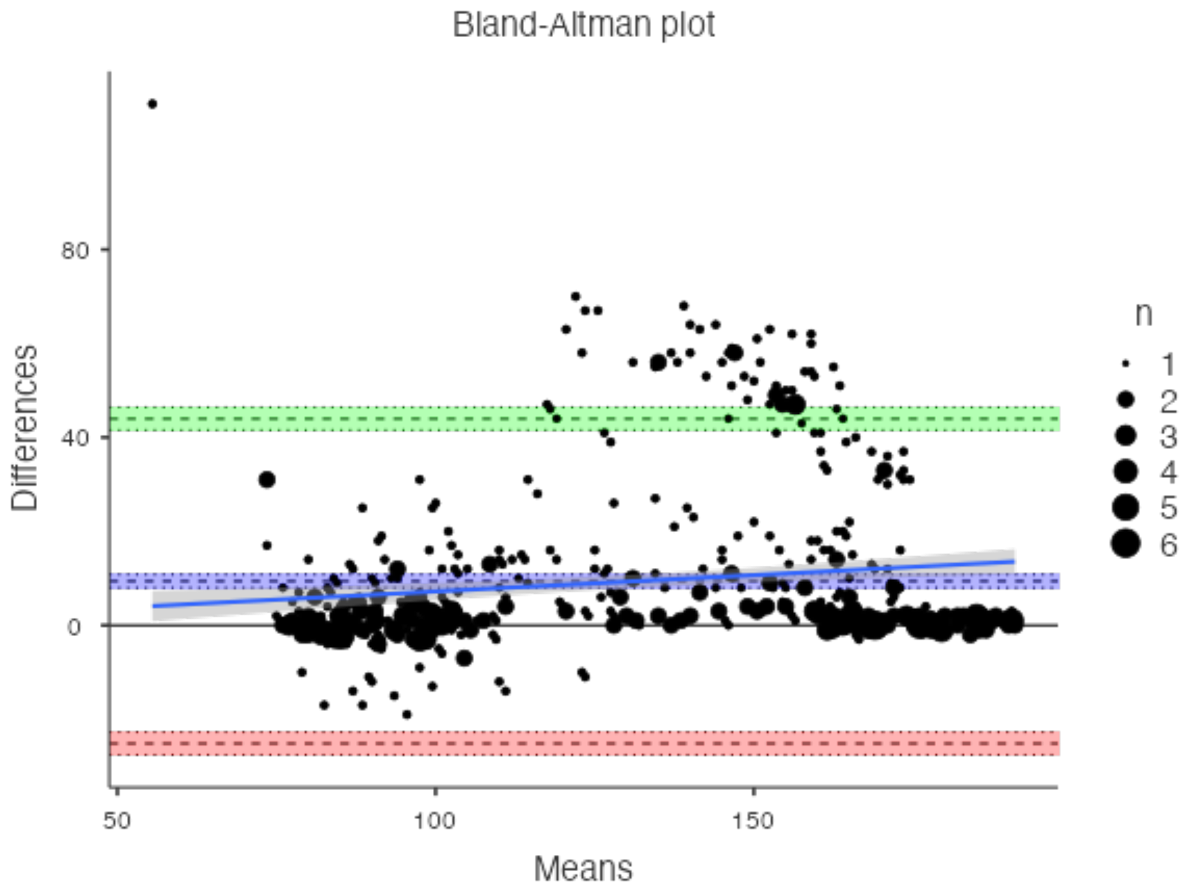

Supplementary Figure S9. Polar Verity Sense 15 second cross-sectional (first 5 minutes only)

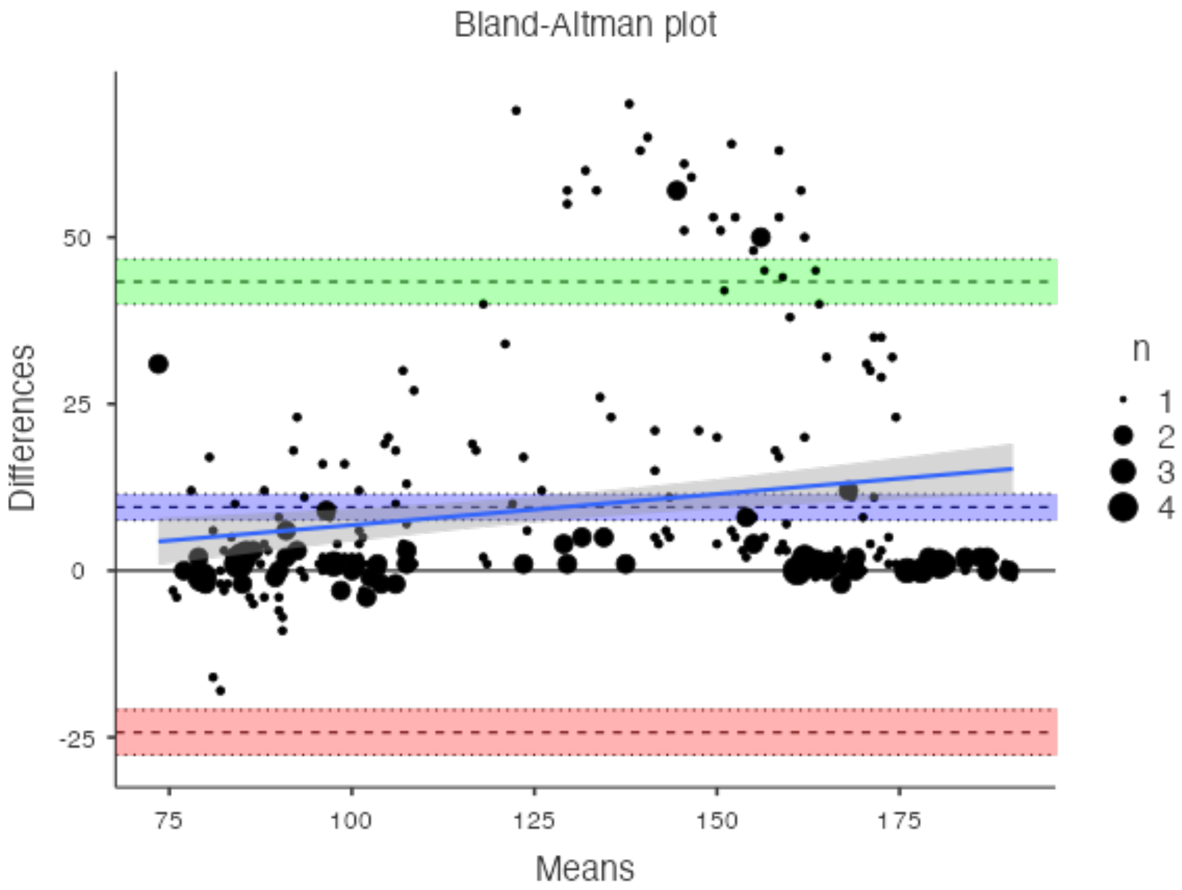

Supplementary Figure S10. Polar Verity Sense 30 second cross-sectional (first 5 minutes only)

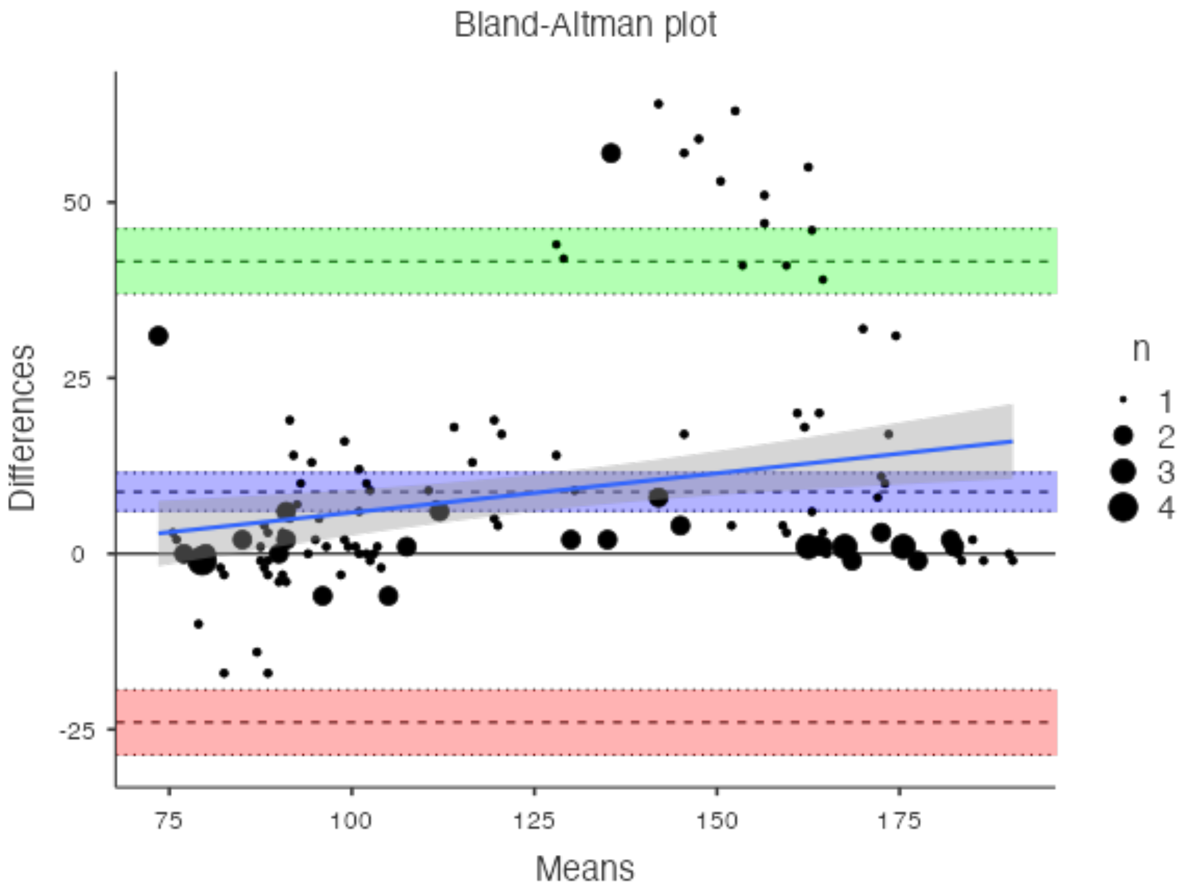

Supplementary Figure S11. Polar Verity Sense 1 minute cross-sectional (first 5 minutes only)

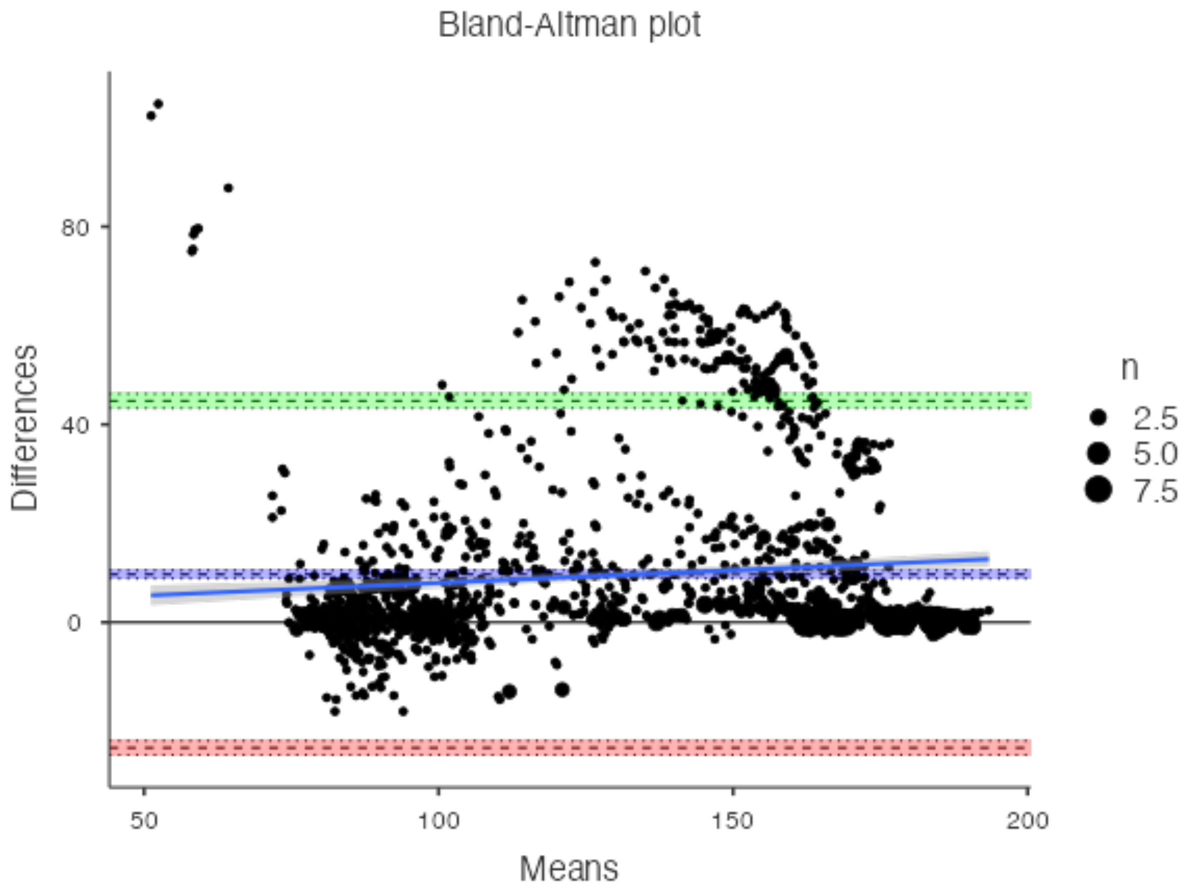

Supplementary Figure S12. Polar Verity Sense 5 second average (first 5 minutes only)

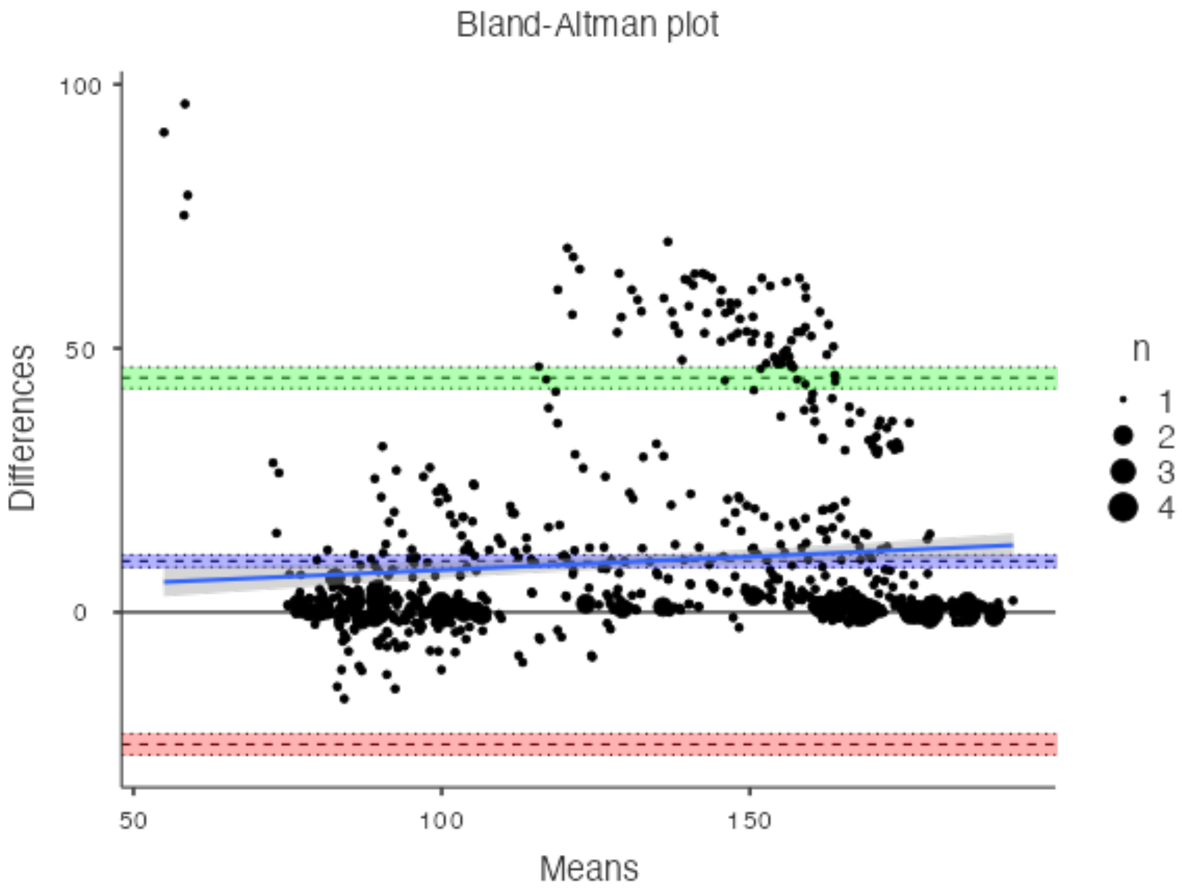

Supplementary Figure S13. Polar Verity Sense 10 second average (first 5 minutes only)

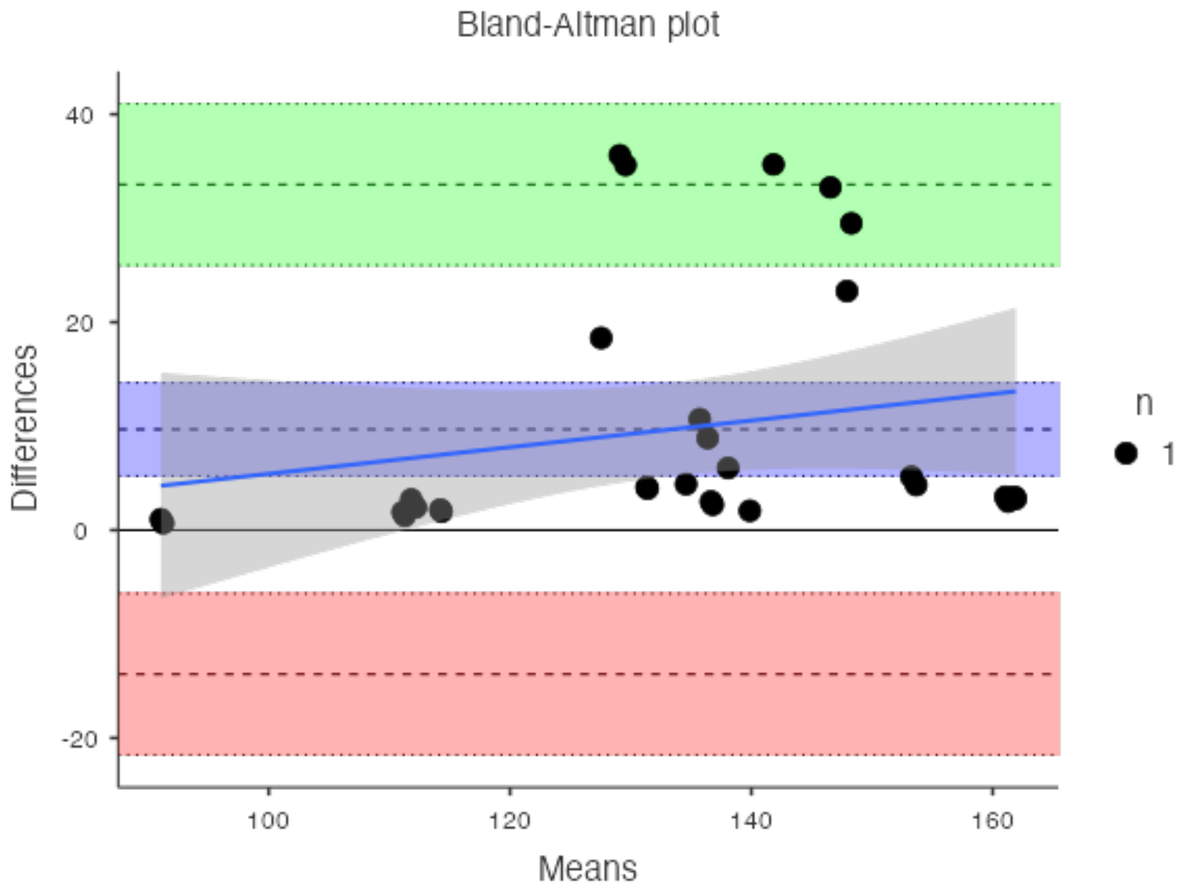

Supplementary Figure S14. Polar Verity Sense session average (first 5 minutes only)

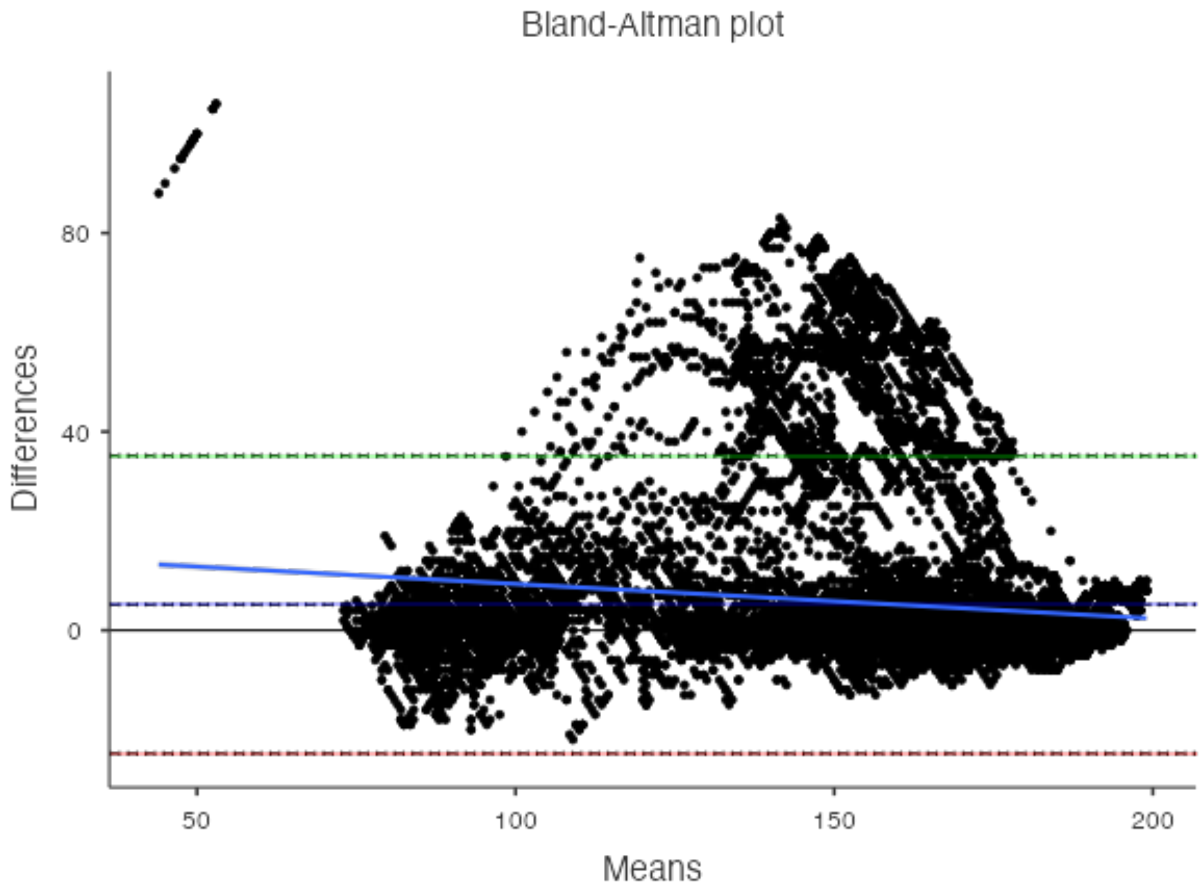

Supplementary Figure S15. Polar OH1 second-by-second (entire trail run)

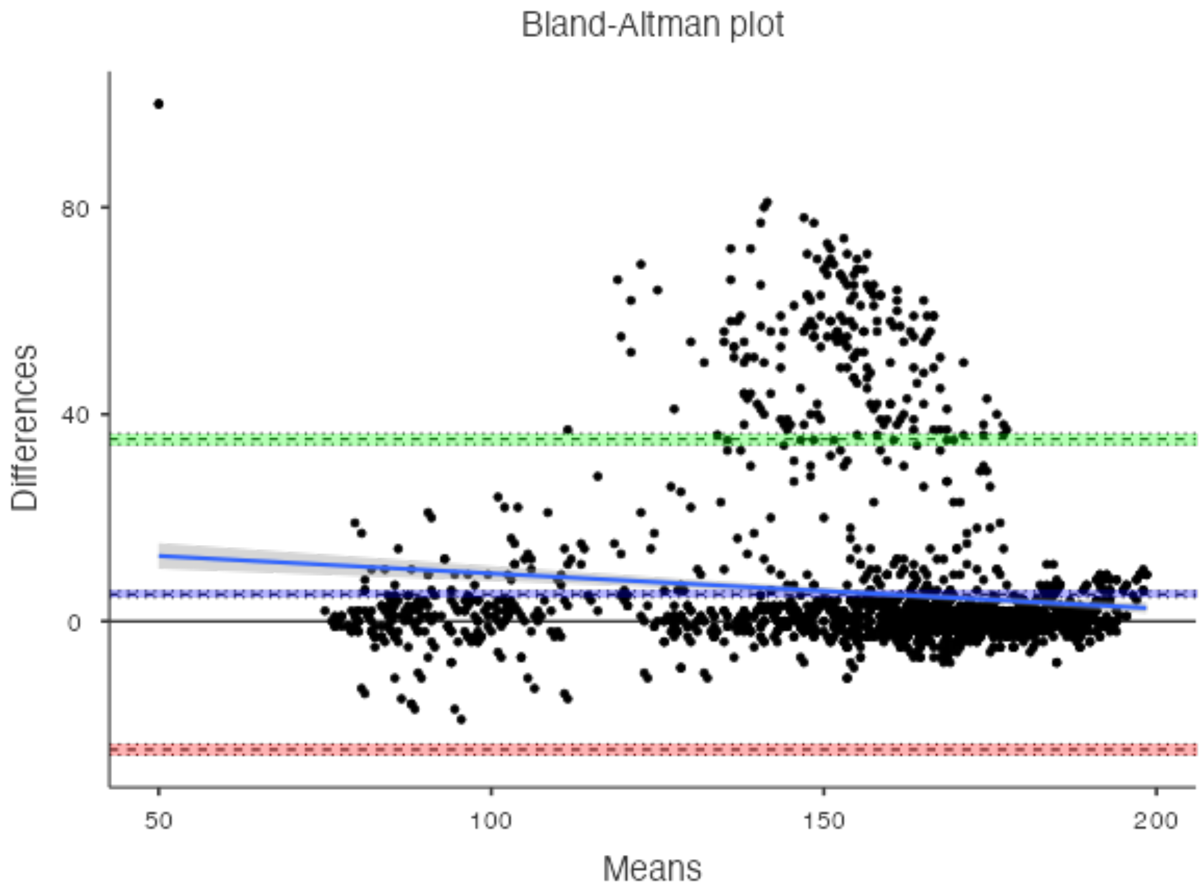

Supplementary Figure S16. Polar OH1 15 second cross-sectional (entire trail run)

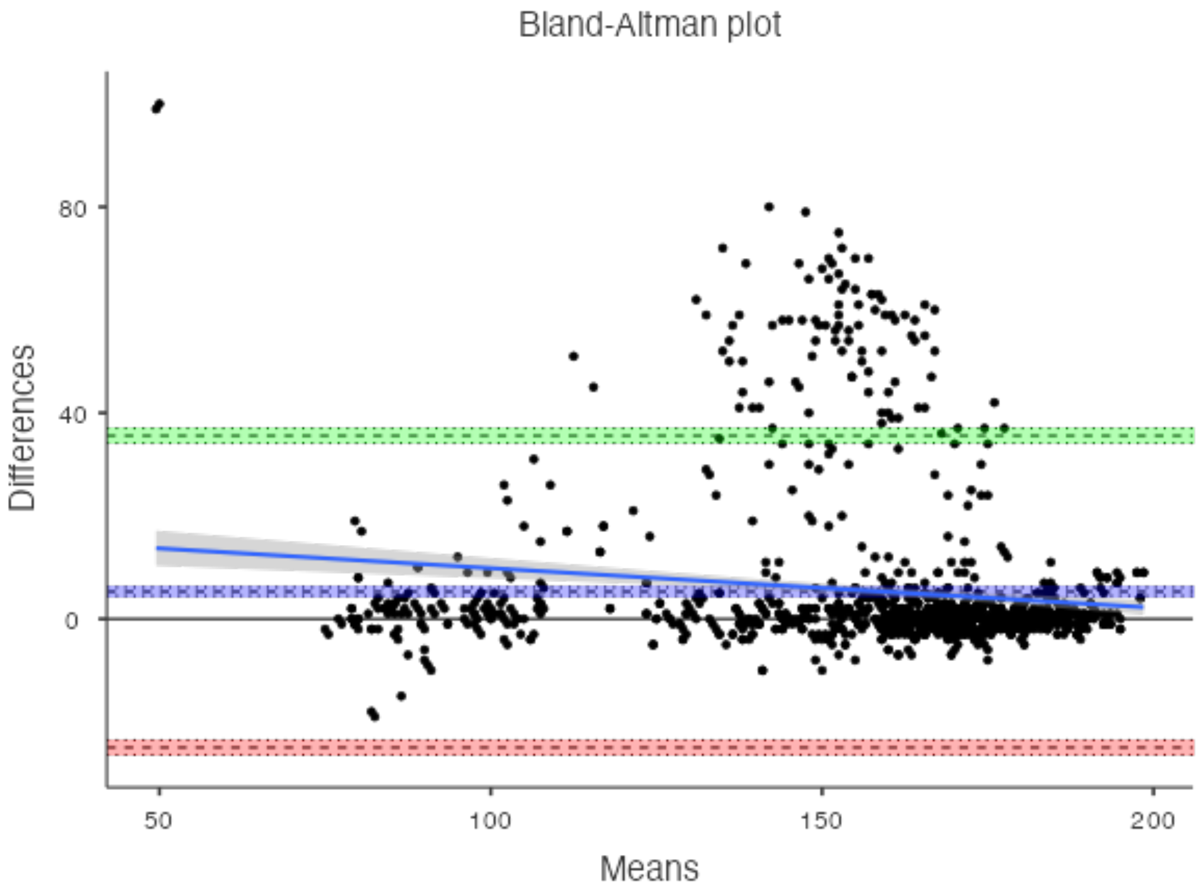

Supplementary Figure S17. Polar OH1 30 second cross-sectional (entire trail run)

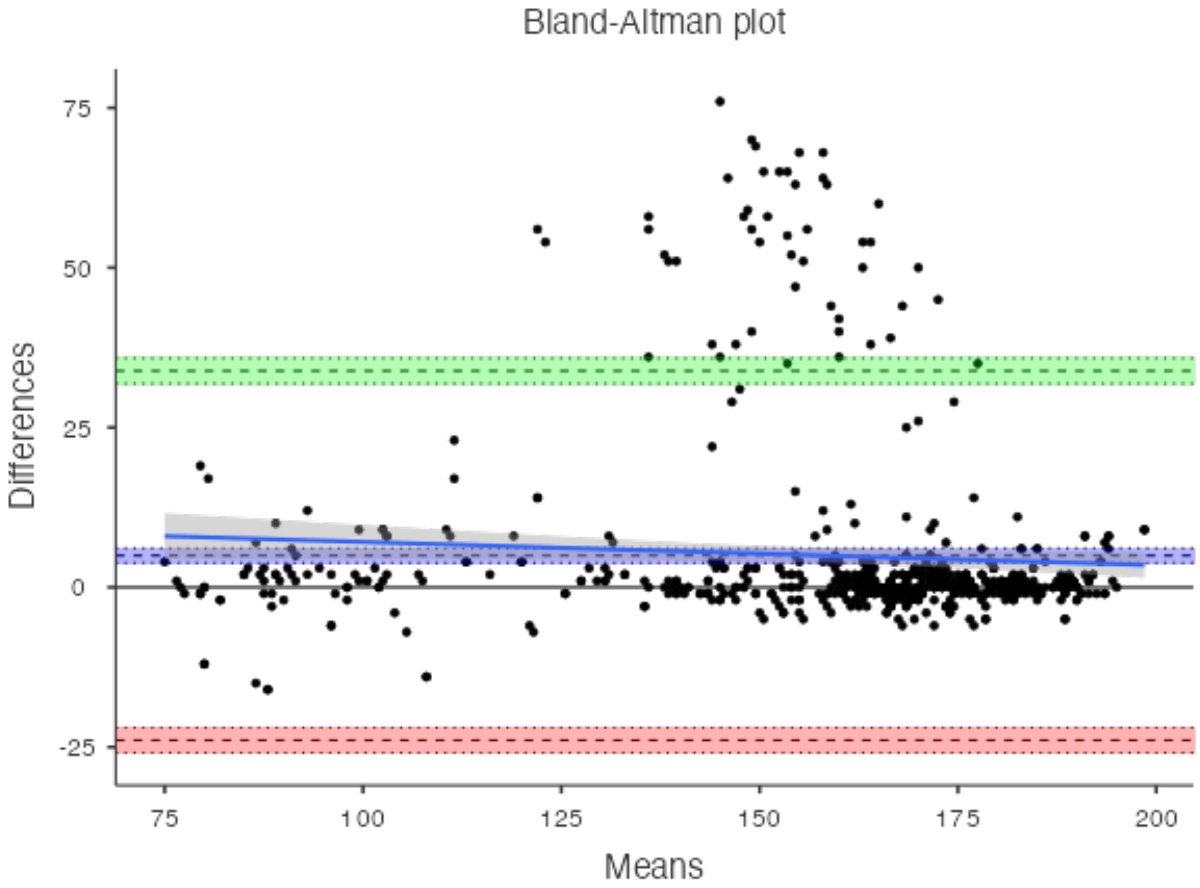

Supplementary Figure S18. Polar OH1 1 minute cross-sectional (entire trail run)

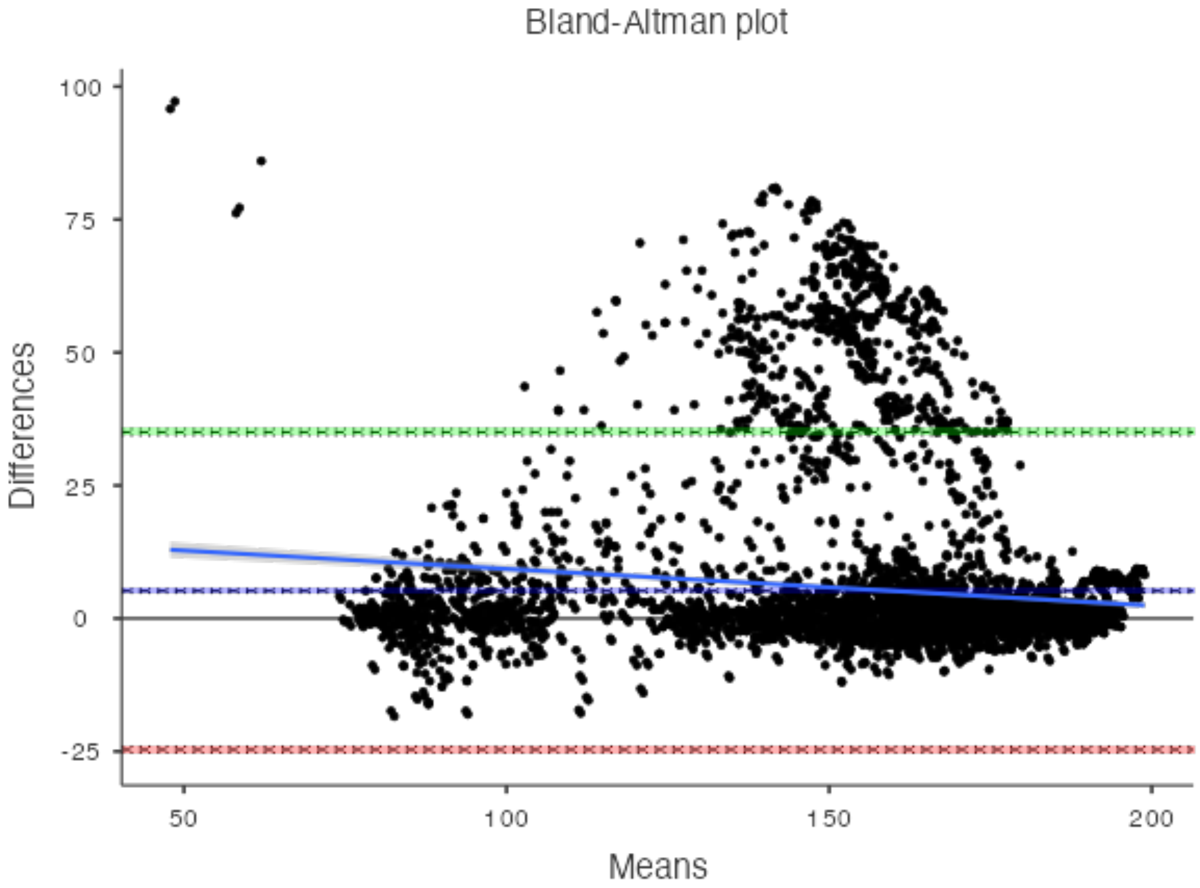

Supplementary Figure S19. Polar OH1 5 second average (entire trail run)

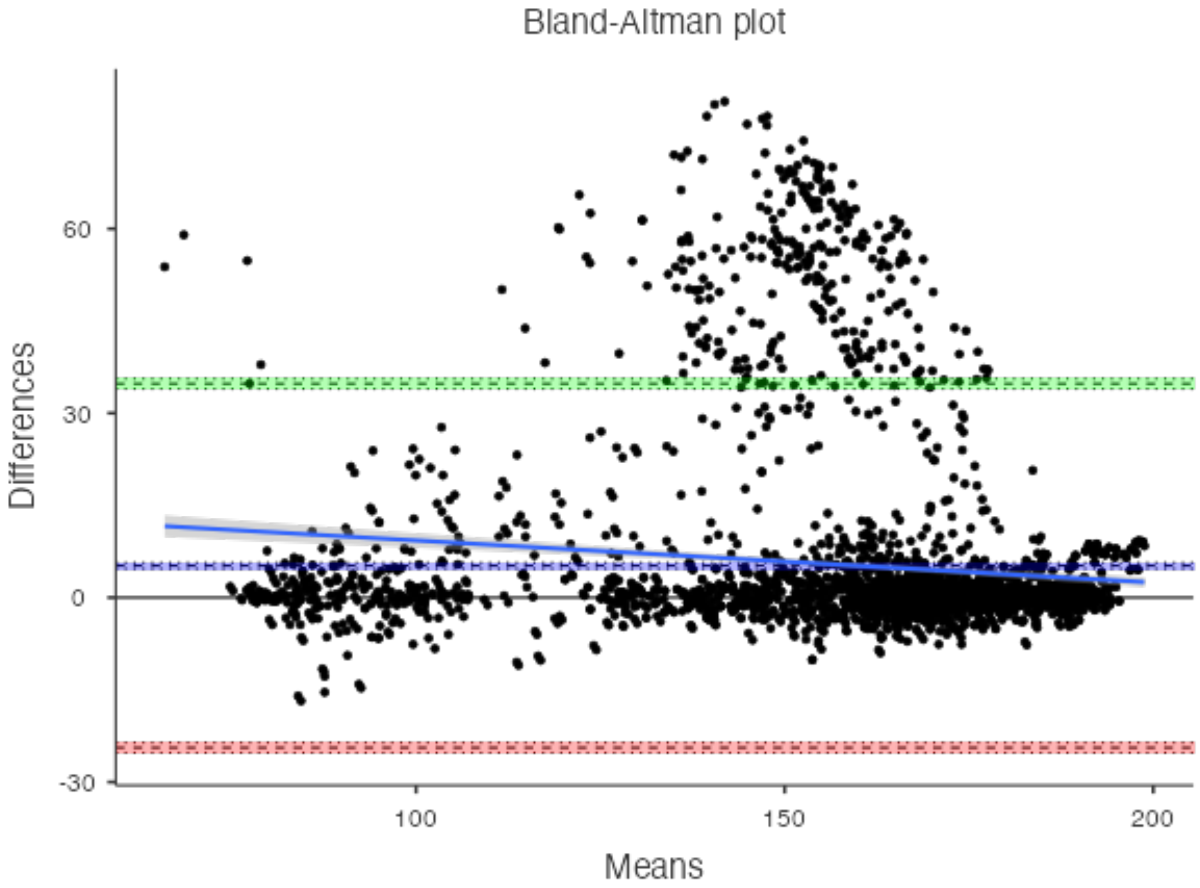

Supplementary Figure S20. Polar OH1 10 second average (entire trail run)

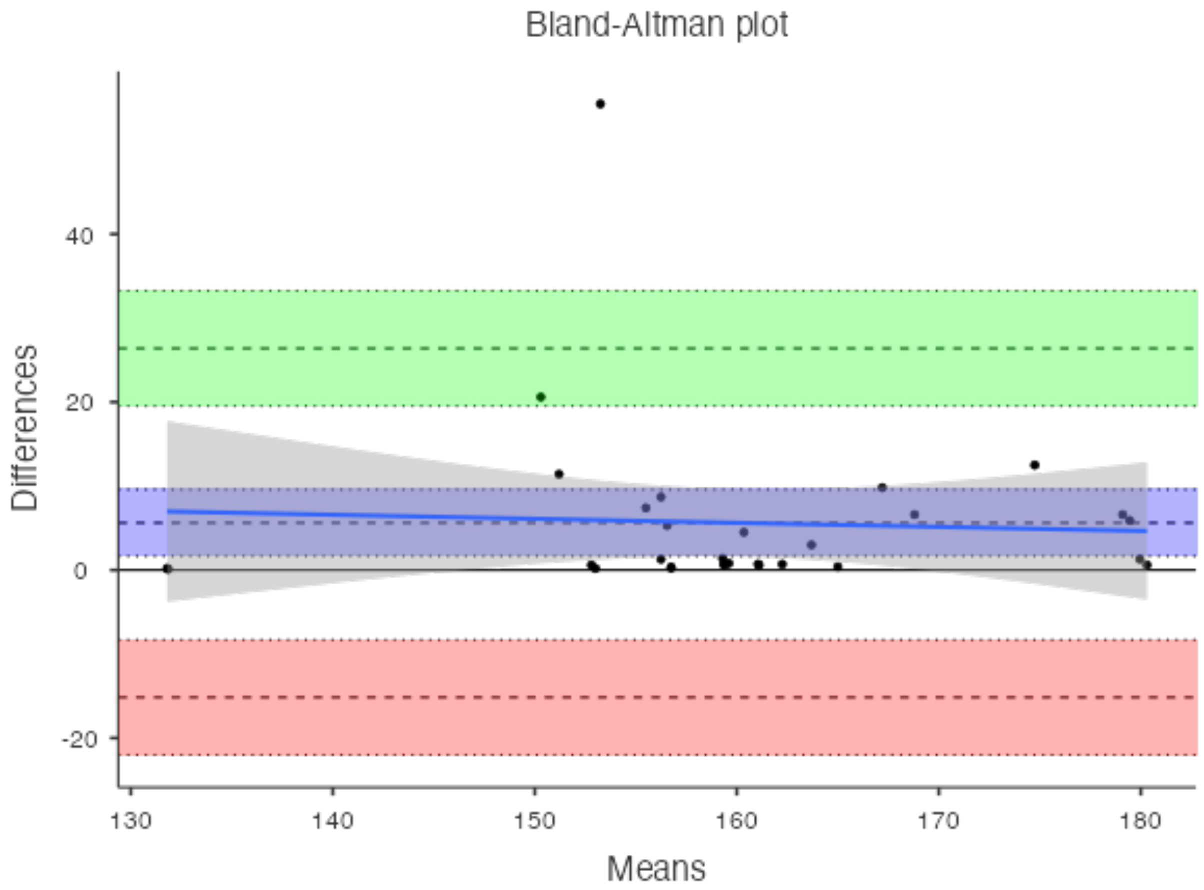

Supplementary Figure S21. Polar OH1 session average (entire trail run)

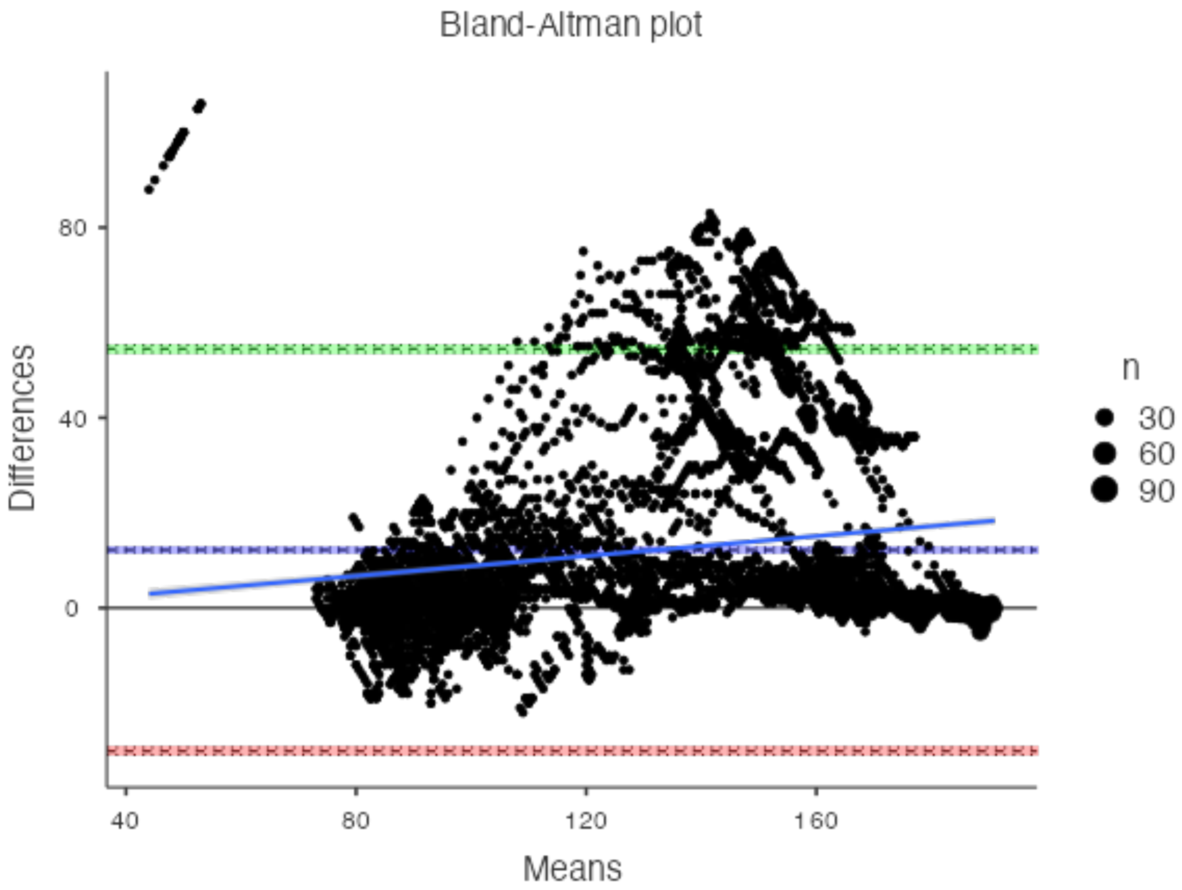

Supplementary Figure S22. Polar OH1 second-by-second (first 5 minutes only)

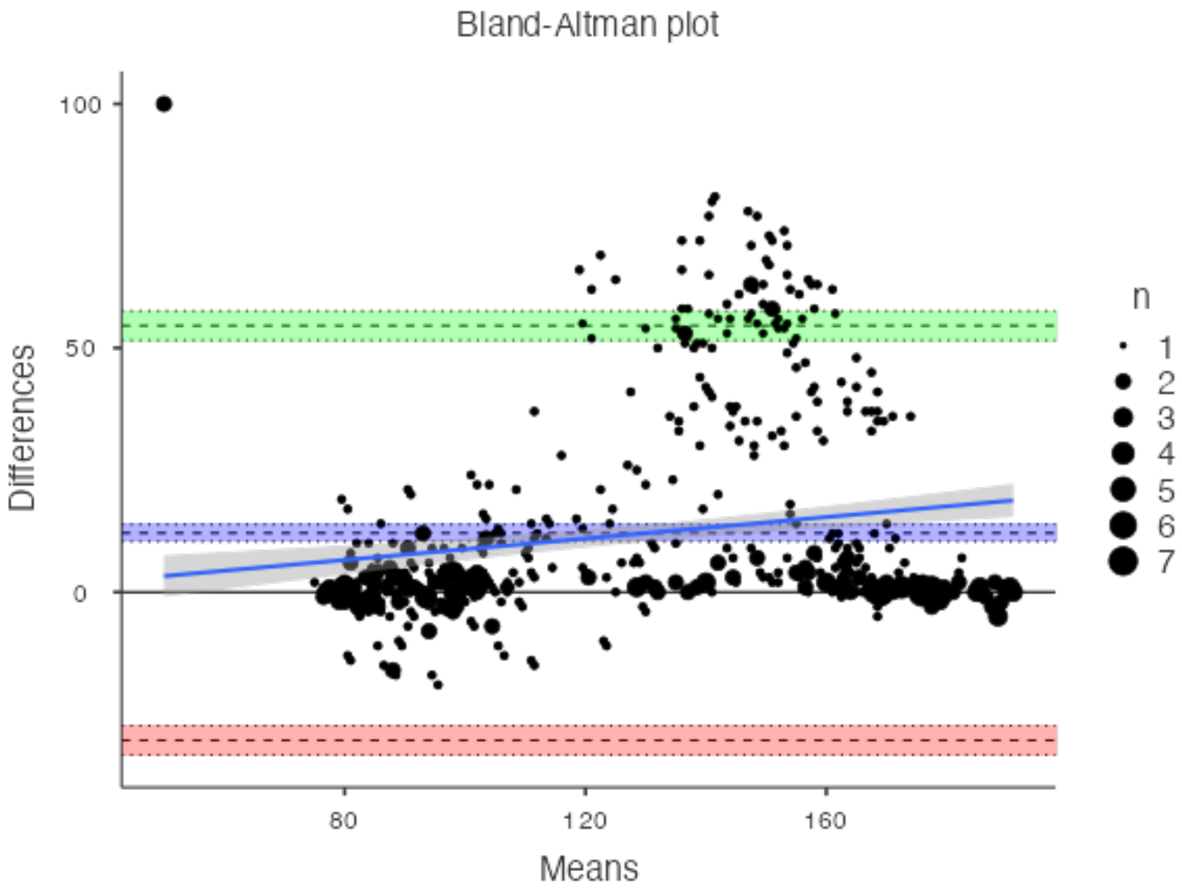

Supplementary Figure S23. Polar OH1 15 second cross-sectional (first 5 minutes only)

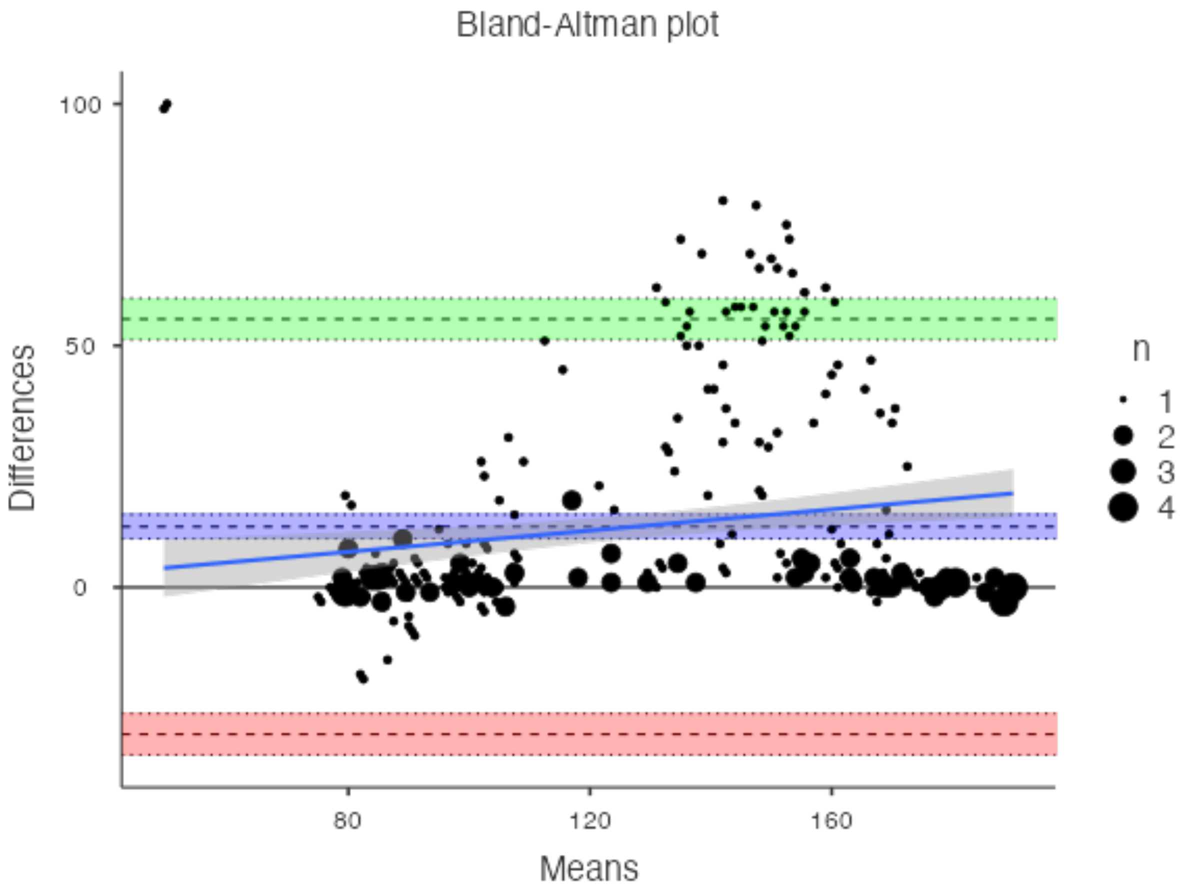

Supplementary Figure S24. Polar OH1 30 second cross-sectional (first 5 minutes only)

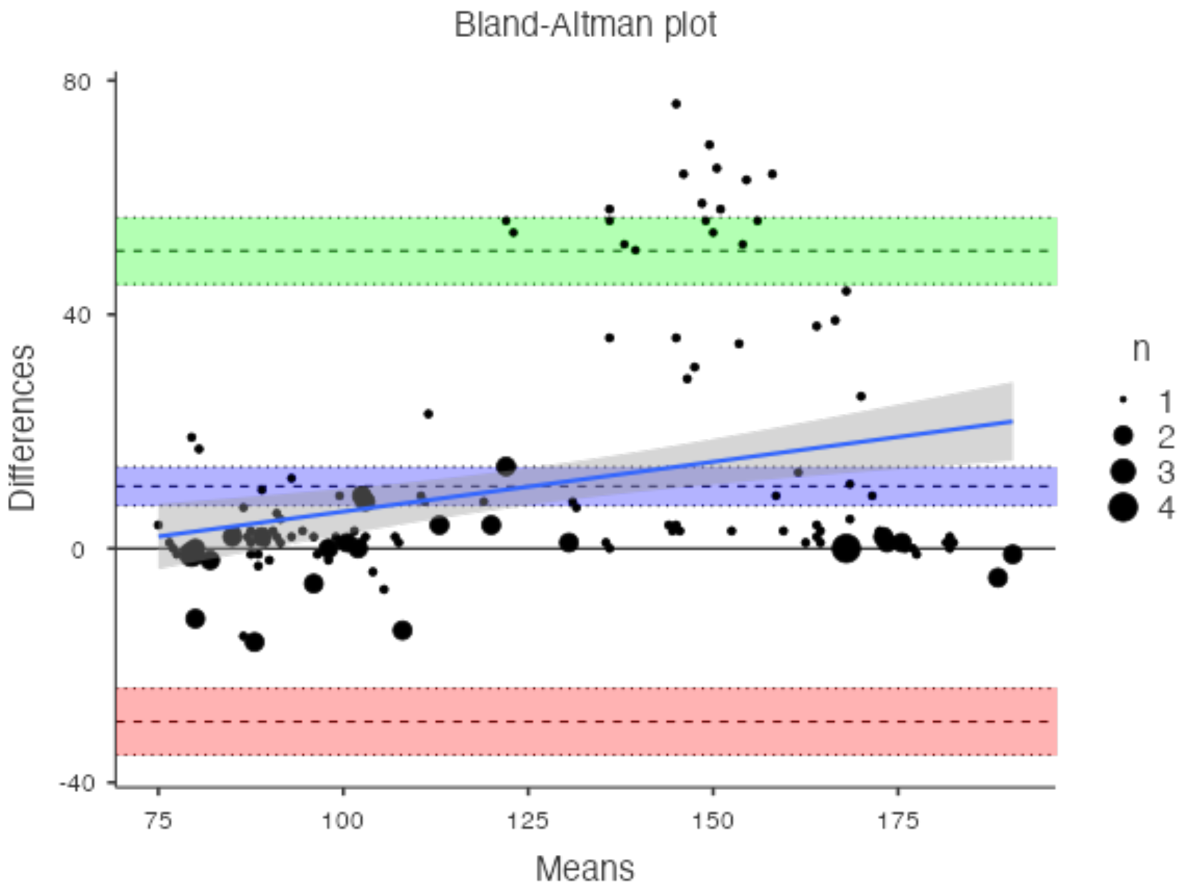

Supplementary Figure S25. Polar OH1 1 minute cross-sectional (first 5 minutes only)

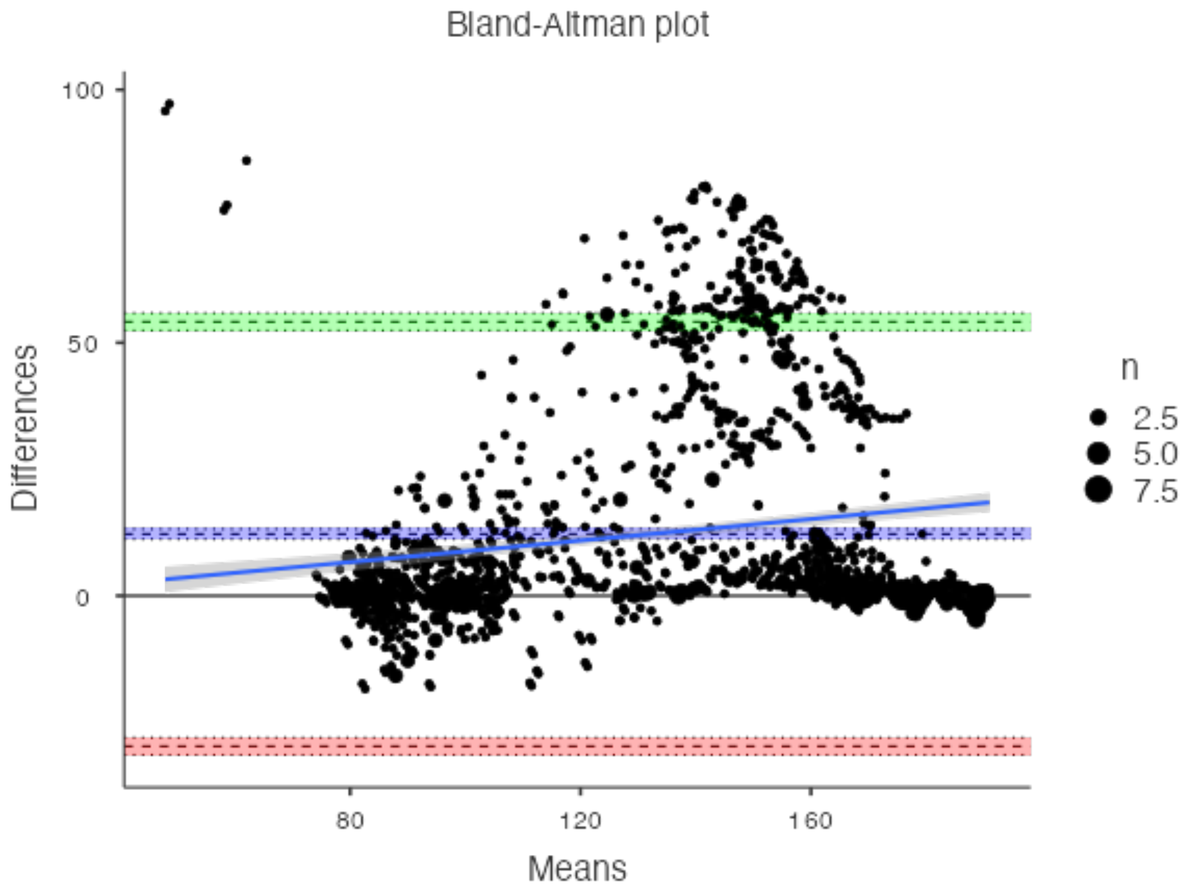

Supplementary Figure S26. Polar OH1 5 second average (first 5 minutes only)

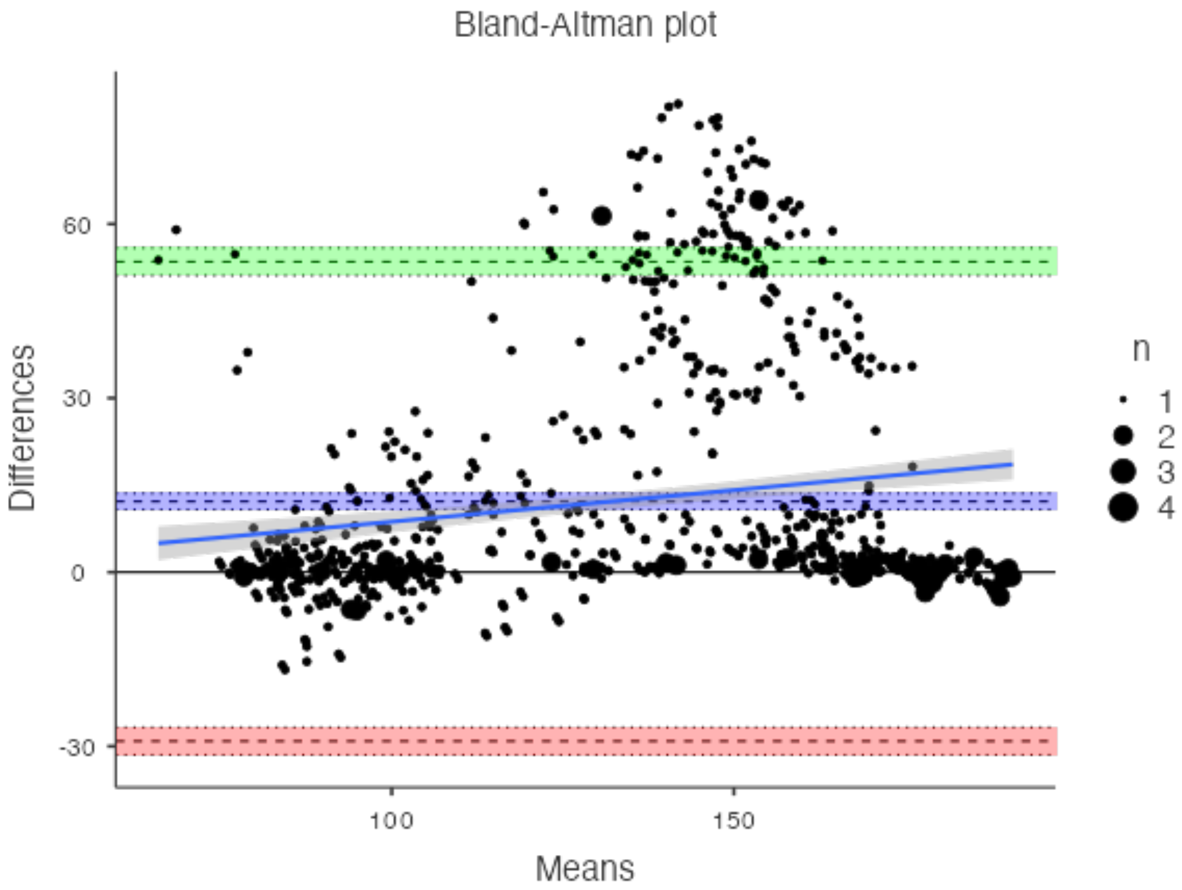

Supplementary Figure S27. Polar OH1 10 second average (first 5 minutes only)

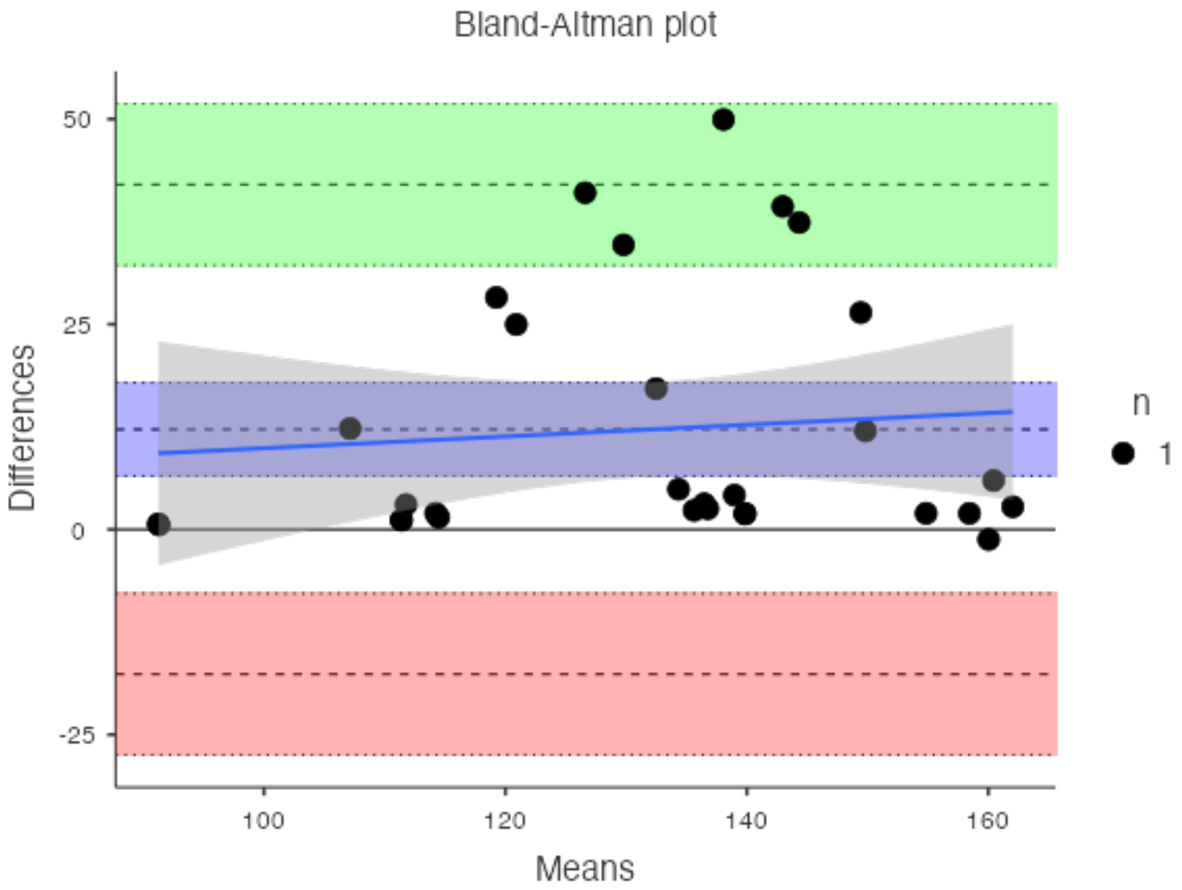

Supplementary Figure S28. Polar OH1 session average (first 5 minutes only)

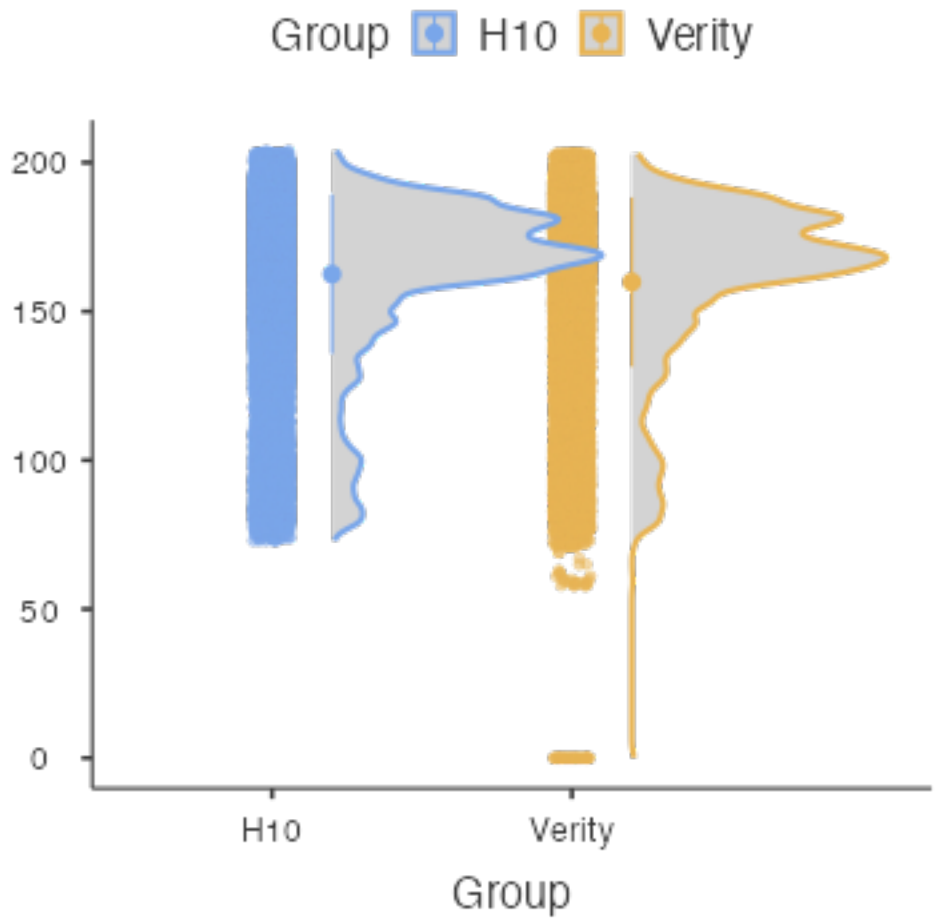

Supplementary Figure S29. Polar Verity Sense second-by-second (entire trail run)

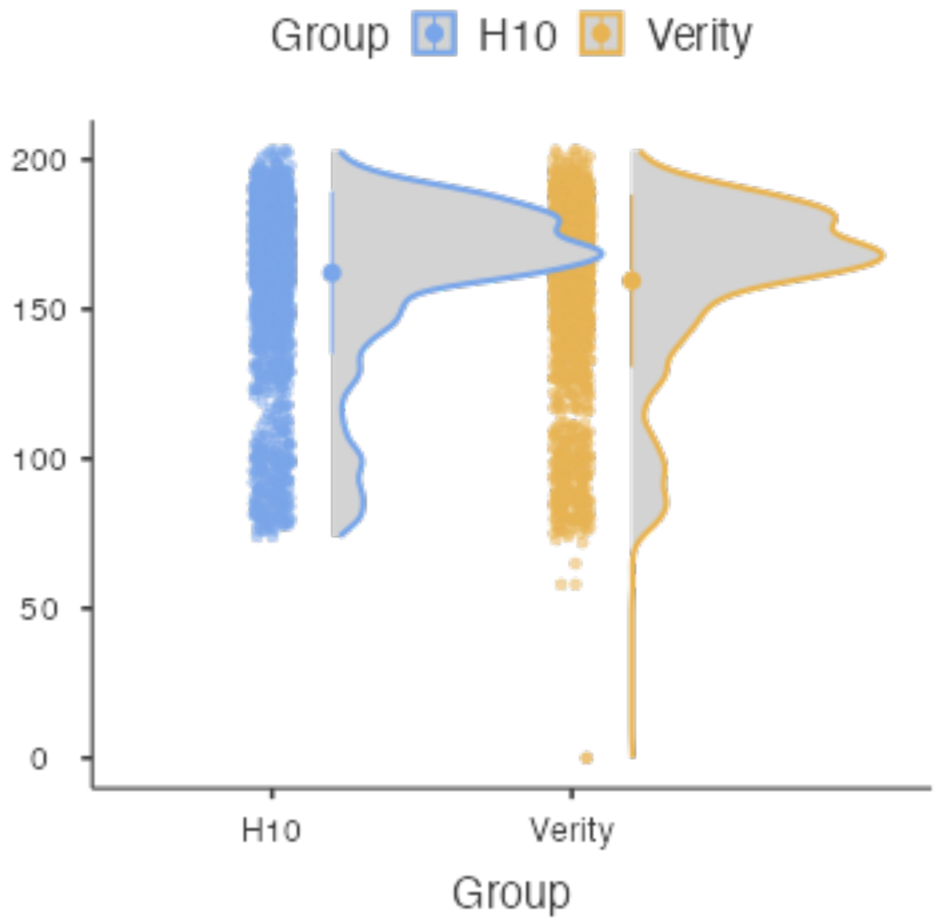

Supplementary Figure S30. Polar Verity Sense 15 second cross-sectional (entire trail run)

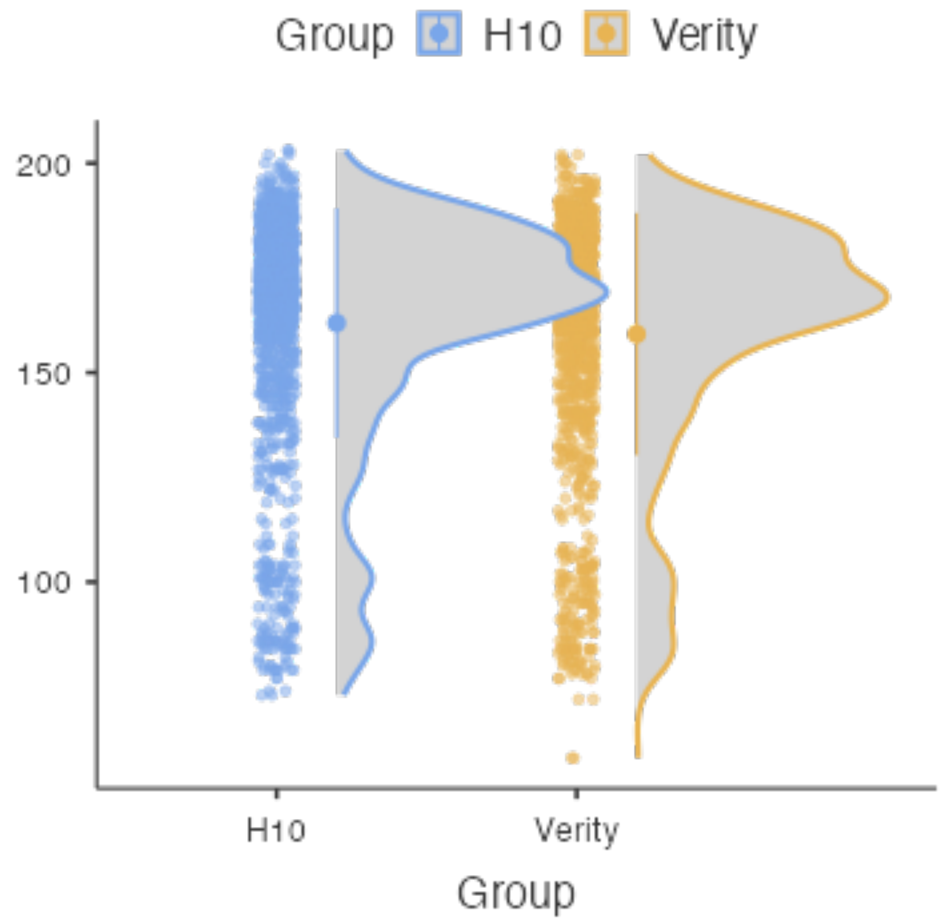

Supplementary Figure S31. Polar Verity Sense 30 second cross-sectional (entire trail run)

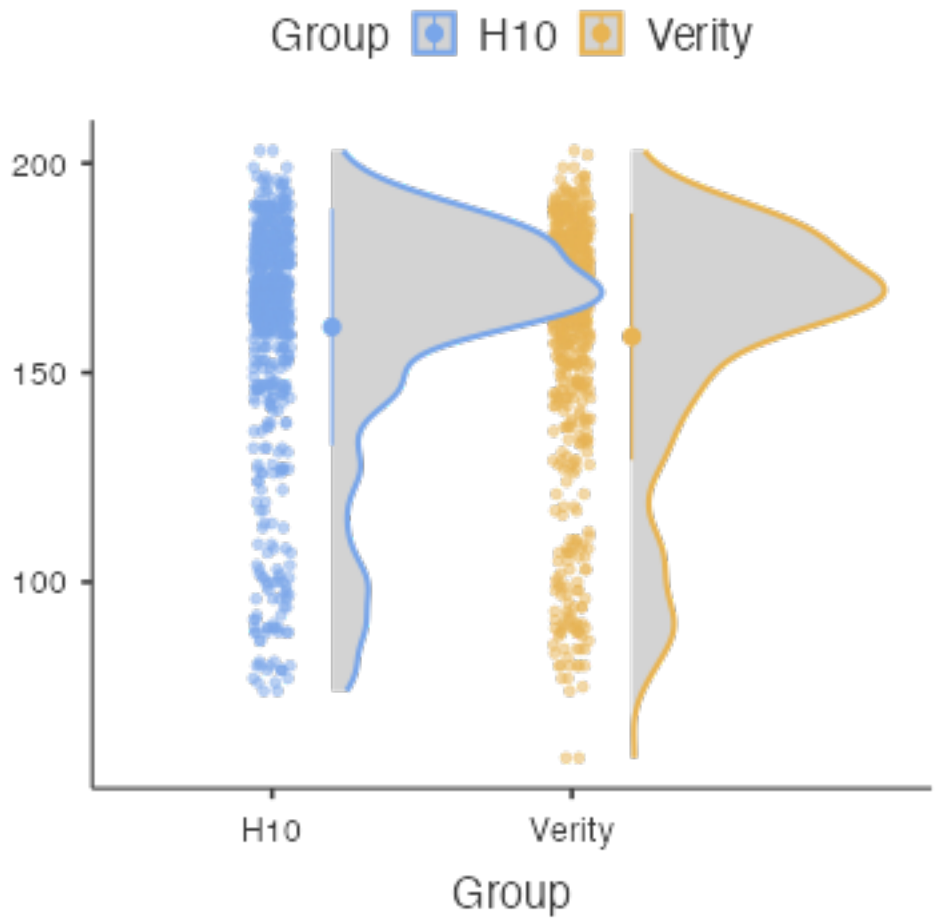

Supplementary Figure S32. Polar Verity Sense 1 minute cross-sectional (entire trail run)

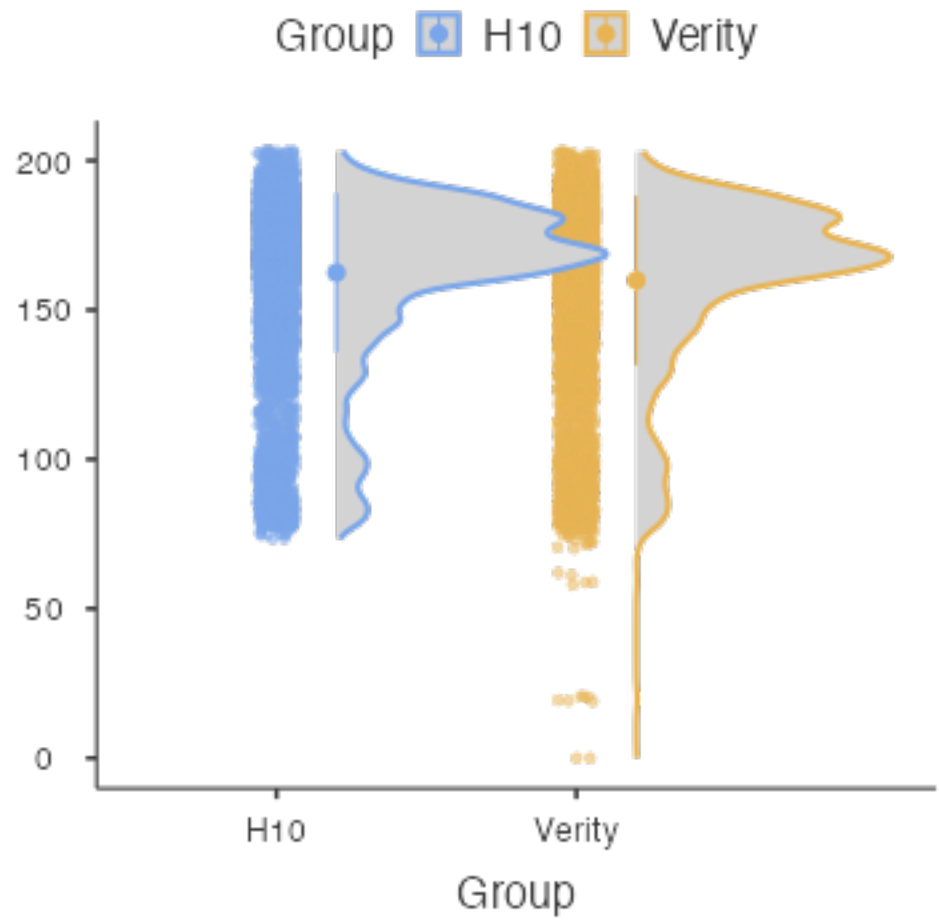

Supplementary Figure S33. Polar Verity Sense 5 second average (entire trail run)

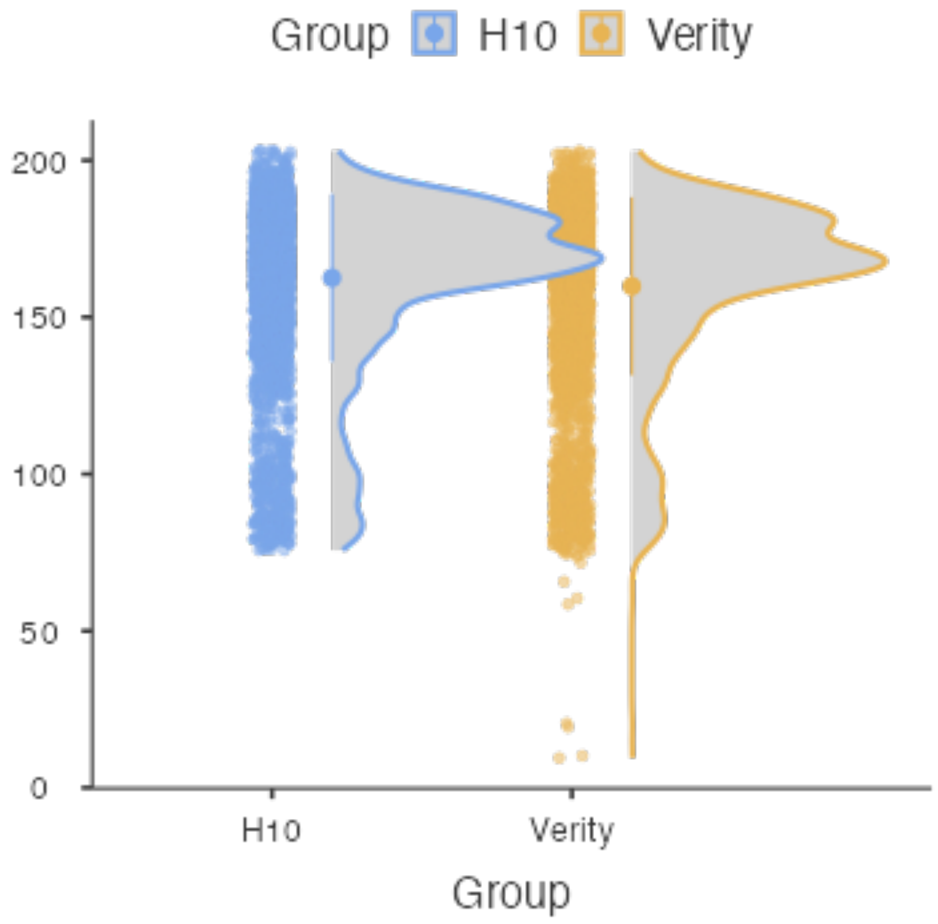

Supplementary Figure S34. Polar Verity Sense 10 second average (entire trail run)

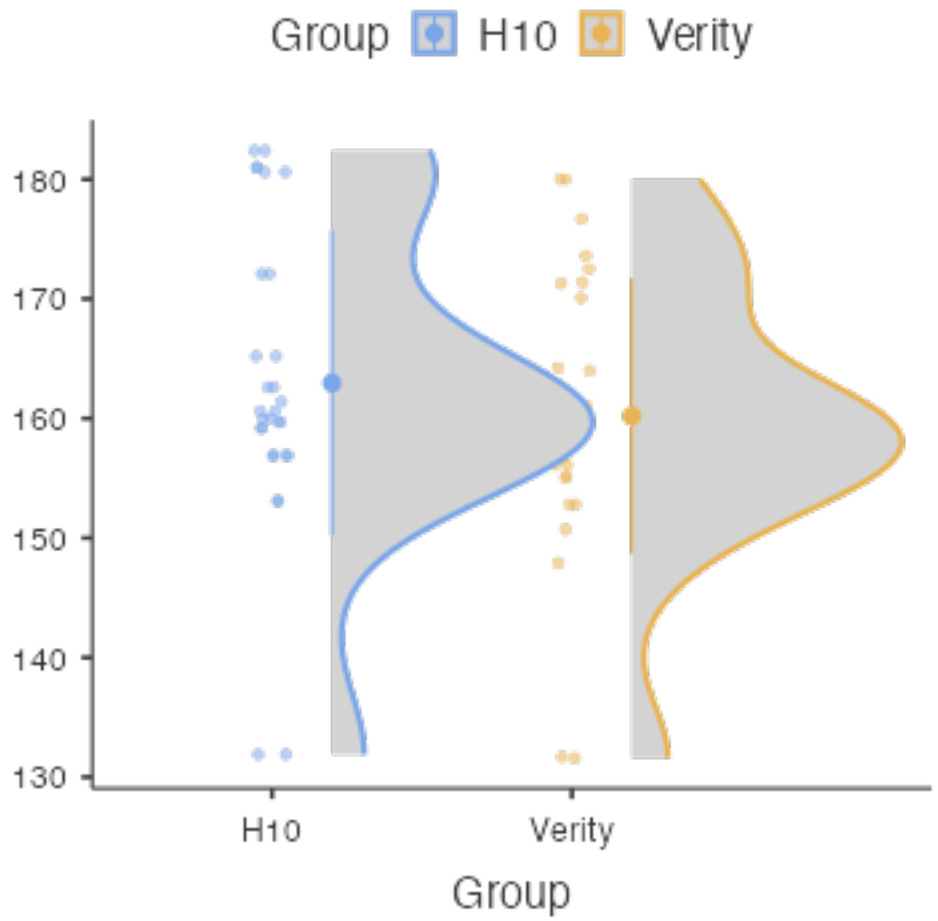

Supplementary Figure S35. Polar Verity Sense session average (entire trail run)

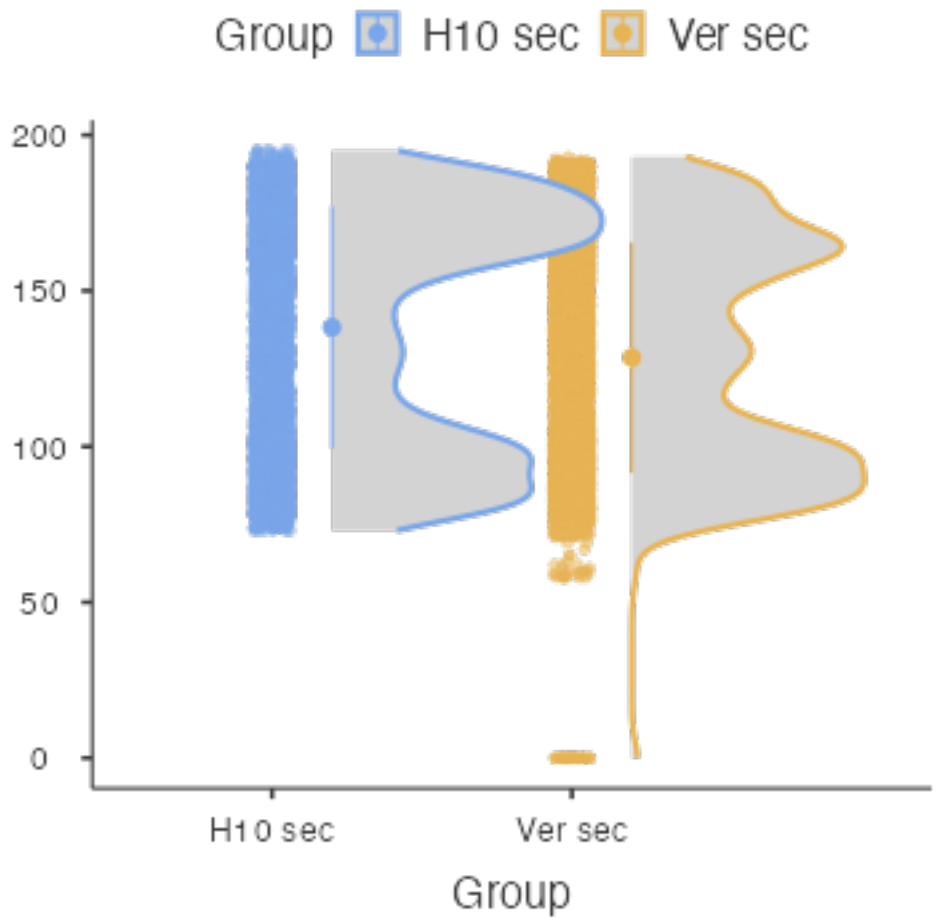

Supplementary Figure S36. Polar Verity Sense second-by-second (first 5 minutes only)

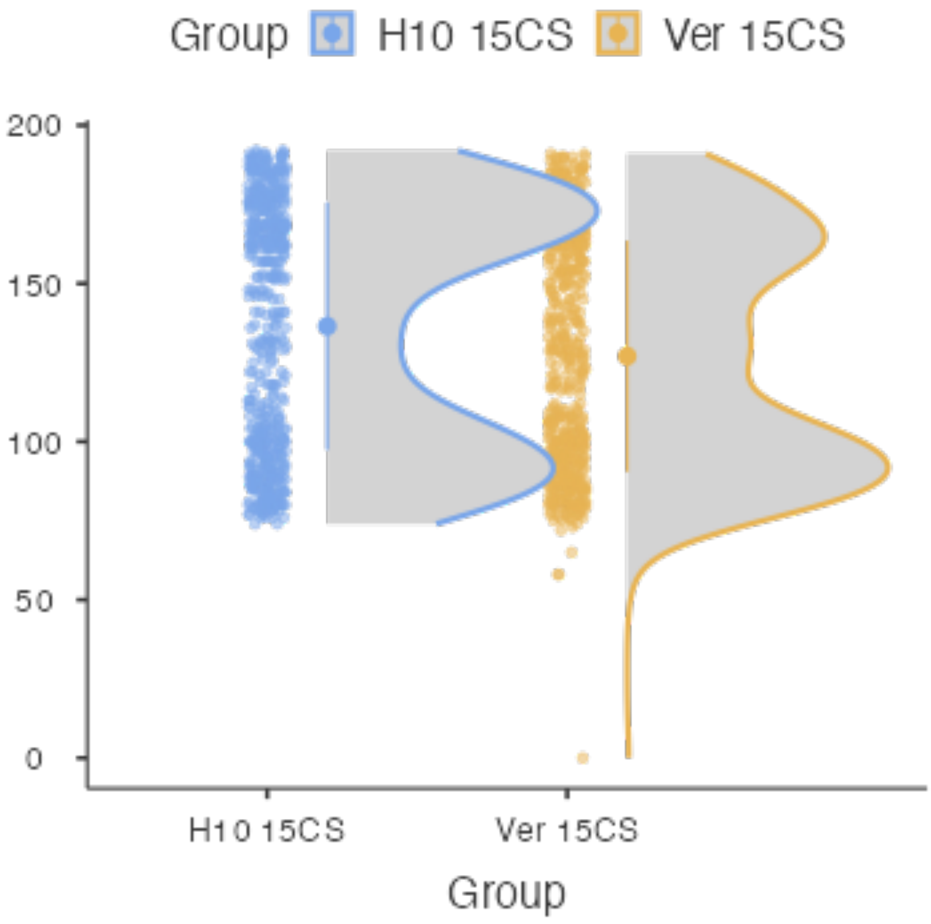

Supplementary Figure S37. Polar Verity Sense 15 second cross-sectional (first 5 minutes only)

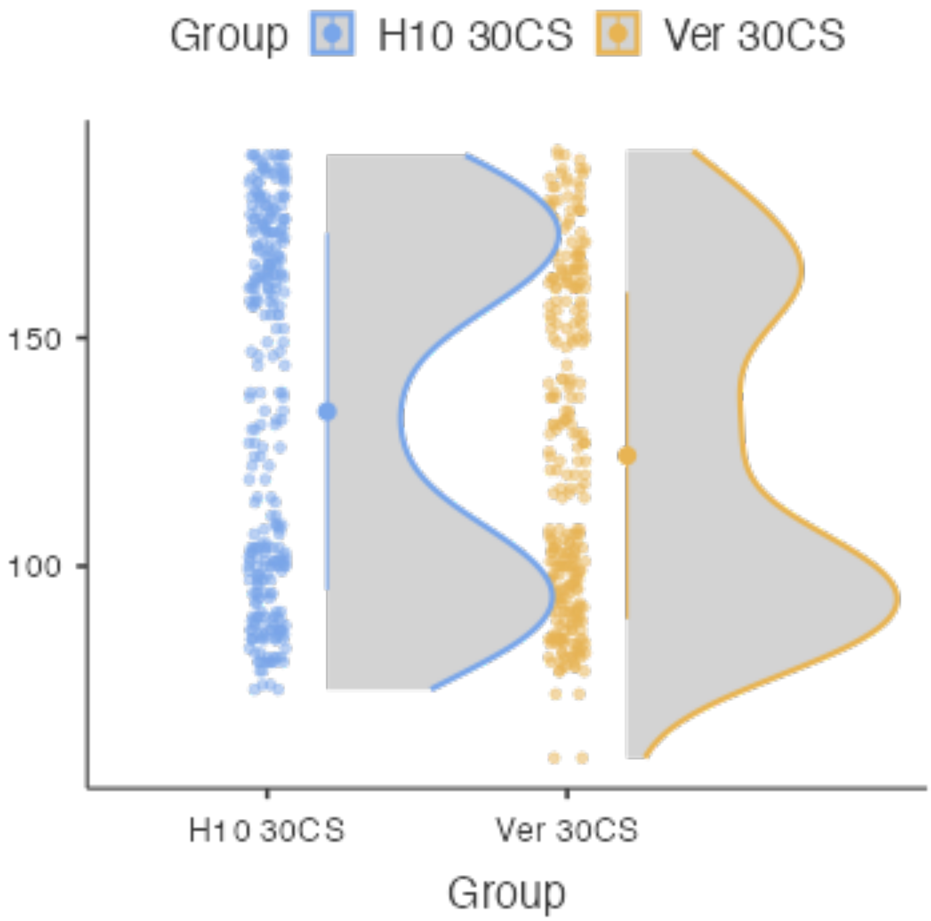

Supplementary Figure S38. Polar Verity Sense 30 second cross-sectional (first 5 minutes only)

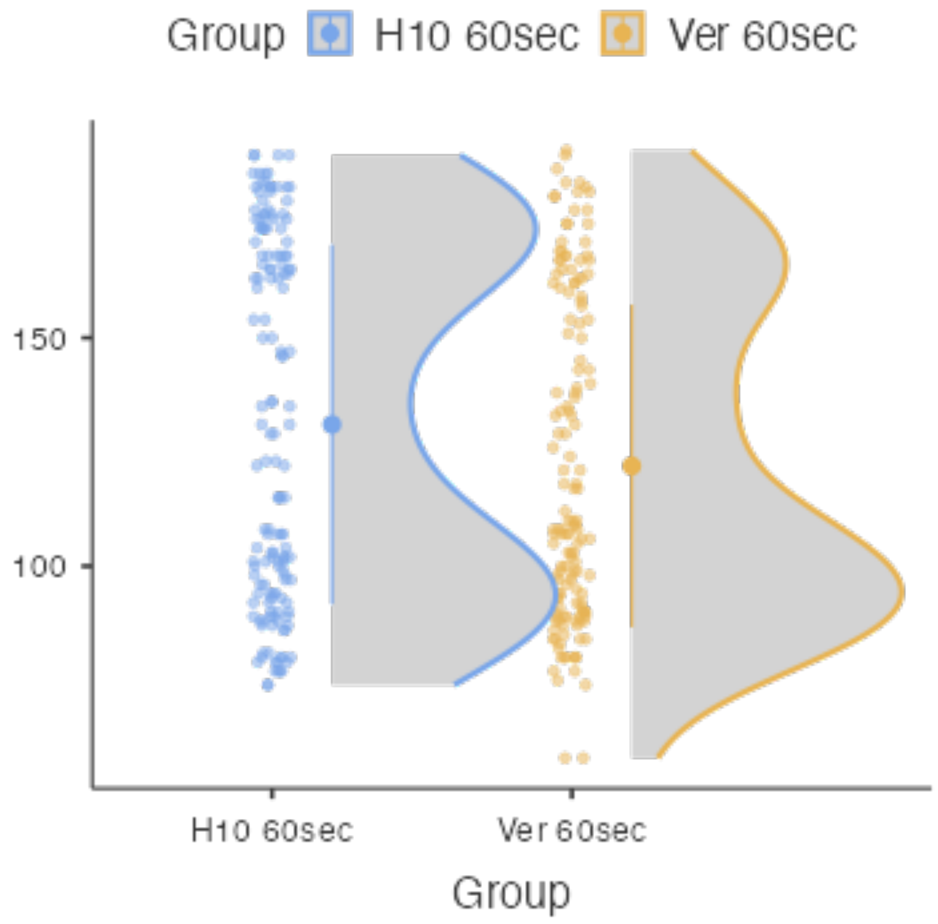

Supplementary Figure S39. Polar Verity Sense 1 minute cross-sectional (first 5 minutes only)

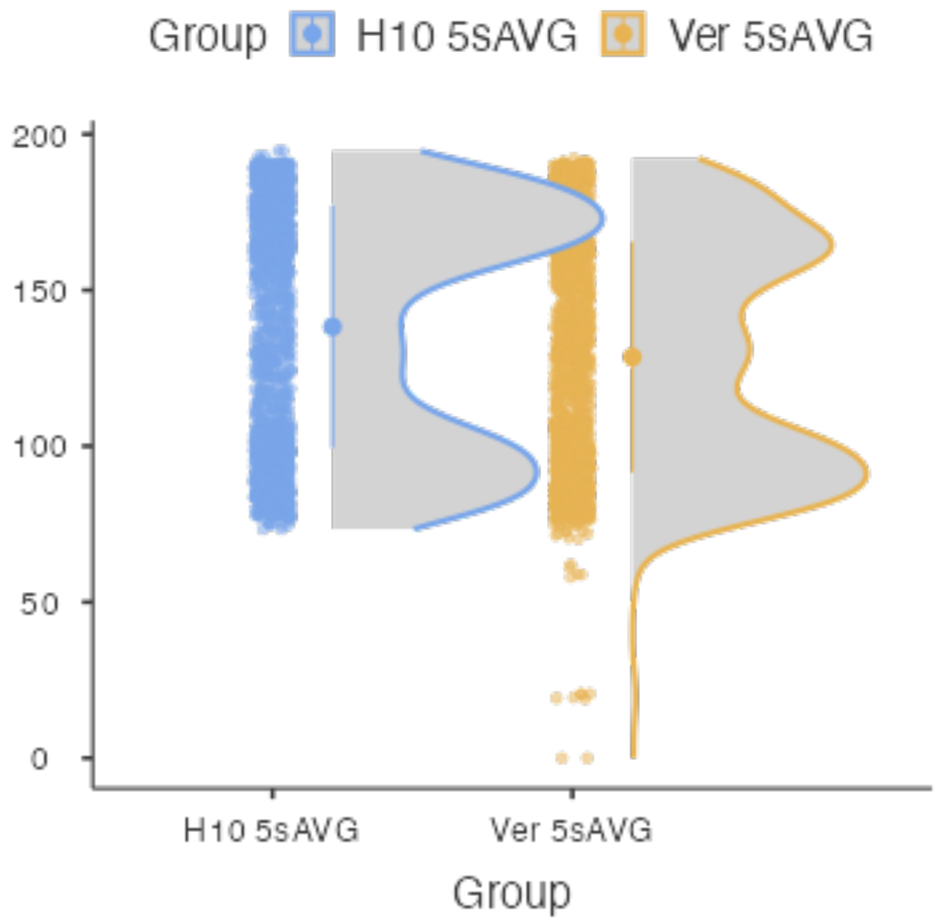

Supplementary Figure S40. Polar Verity Sense 5 second average (first 5 minutes only)

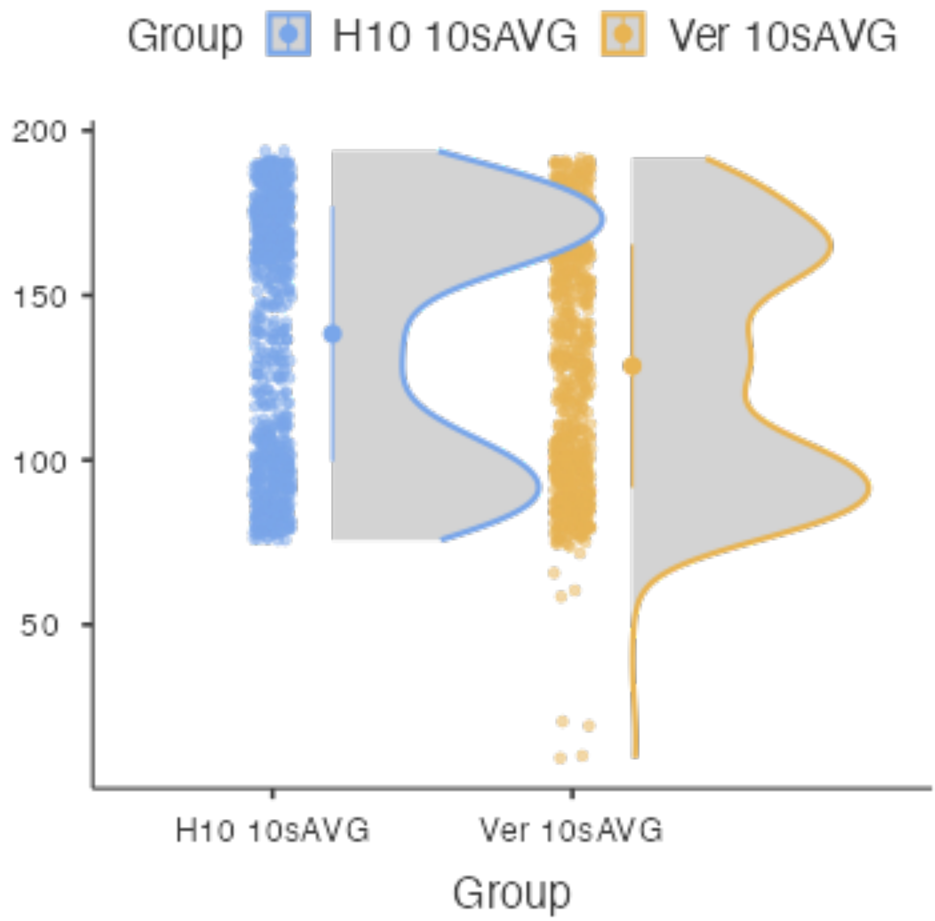

Supplementary Figure S41. Polar Verity Sense 10 second average (first 5 minutes only)

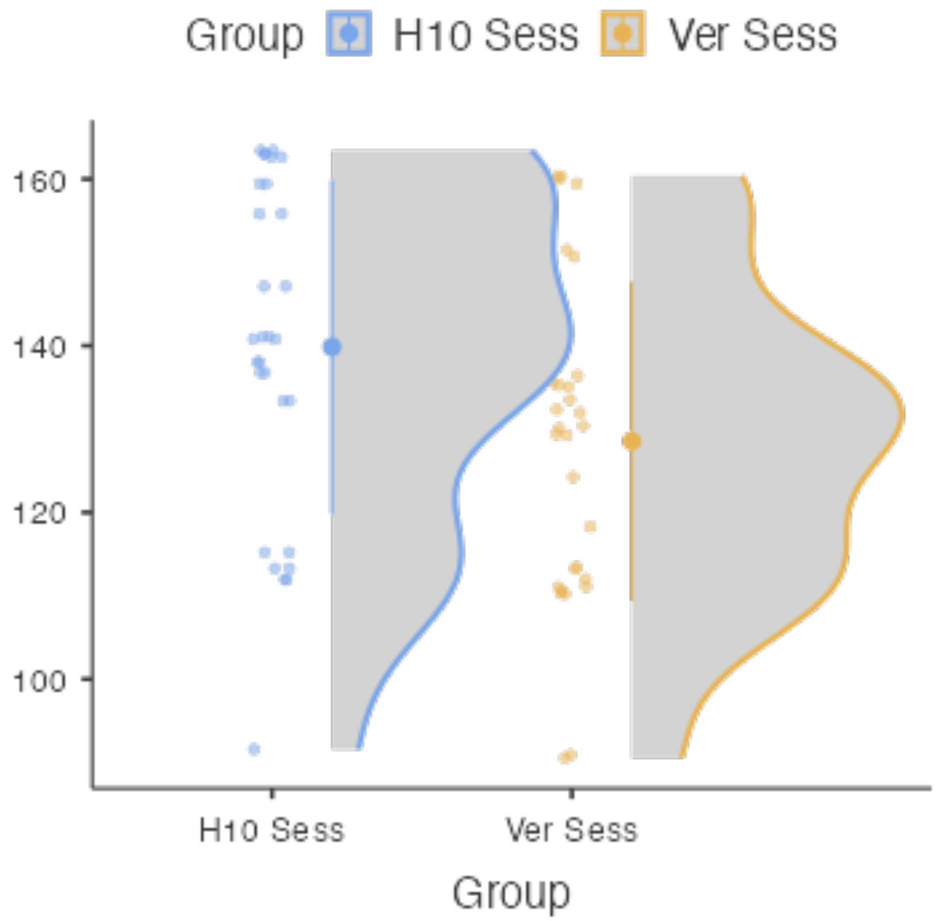

Supplementary Figure S42. Polar Verity Sense session average (first 5 minutes only)

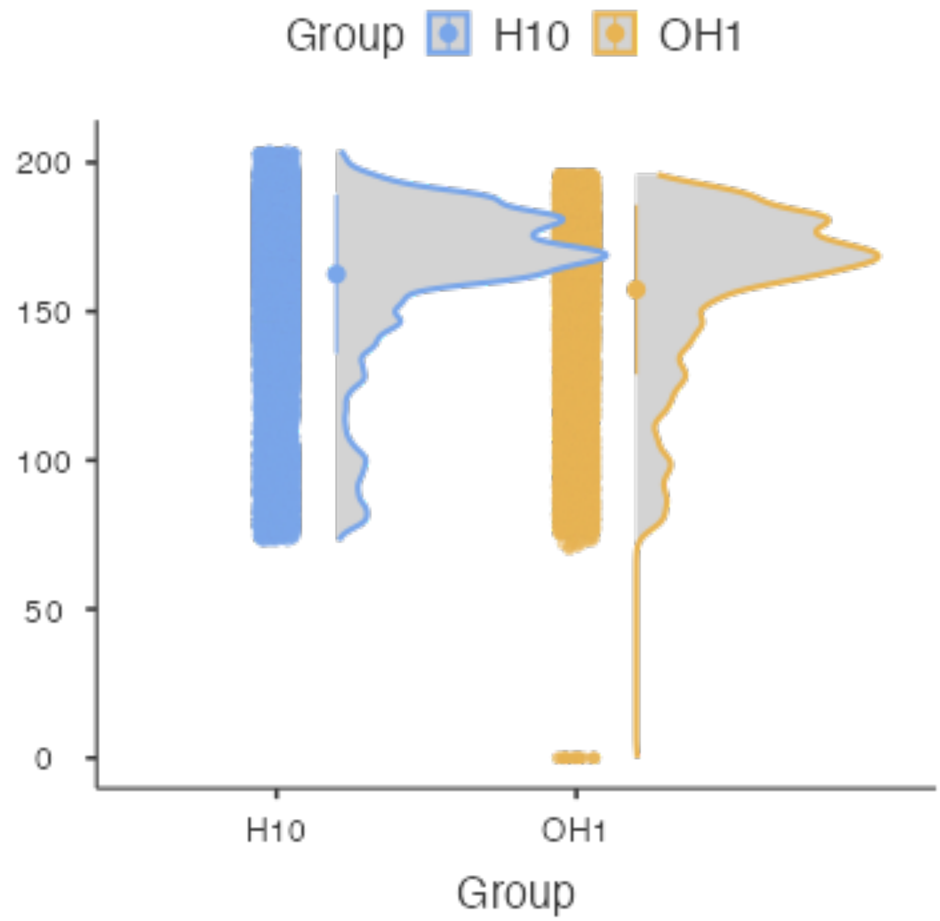

Supplementary Figure S43. Polar OH1 second-by-second (entire trail run)

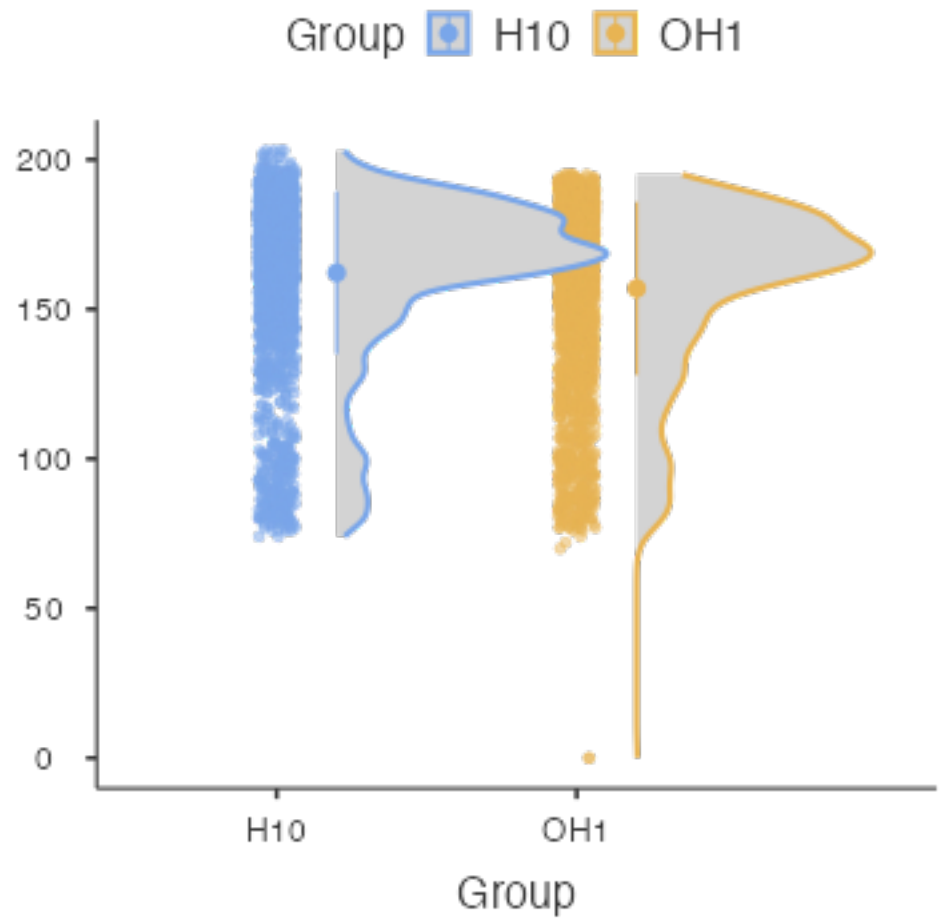

Supplementary Figure S44. Polar OH1 15 second cross-sectional (entire trail run)

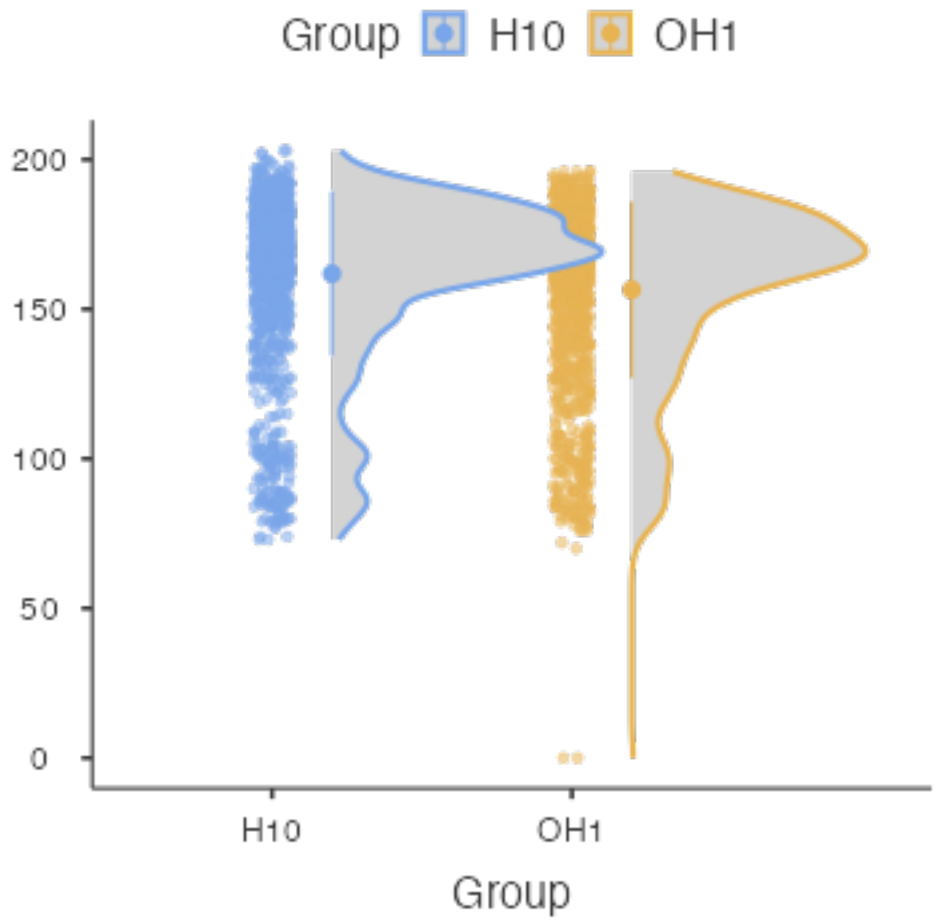

Supplementary Figure S45. Polar OH1 30 second cross-sectional (entire trail run)

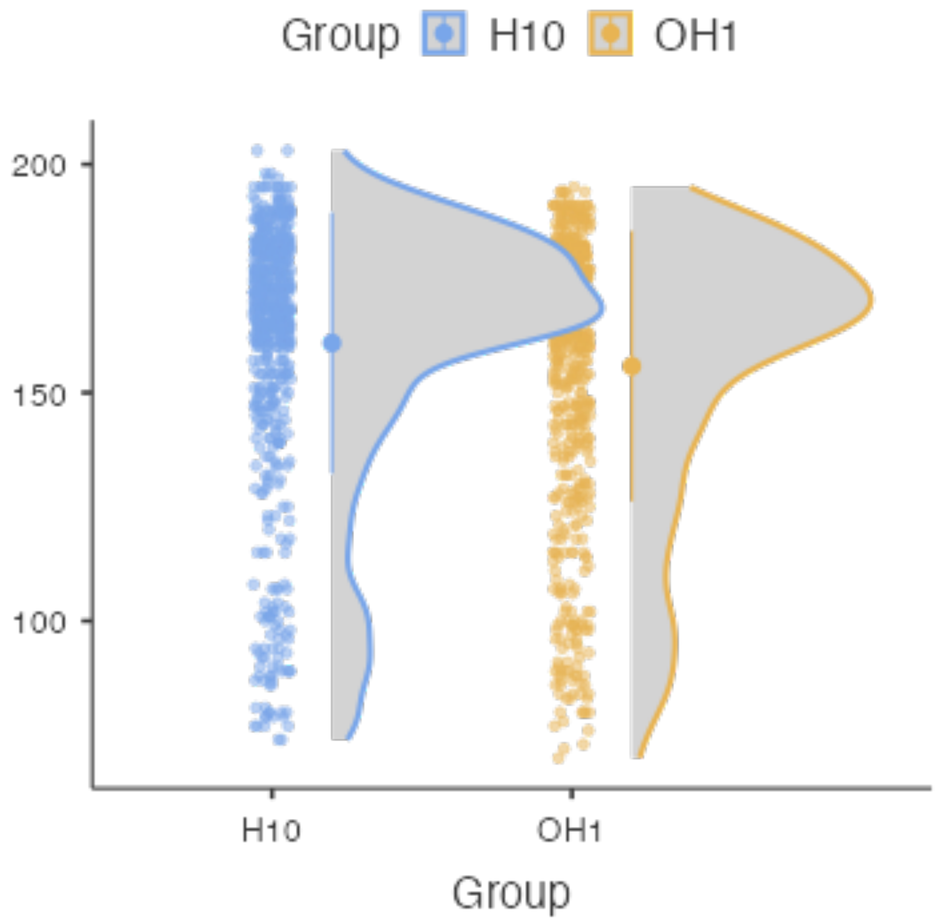

Supplementary Figure S46. Polar OH1 1 minute cross-sectional (entire trail run)

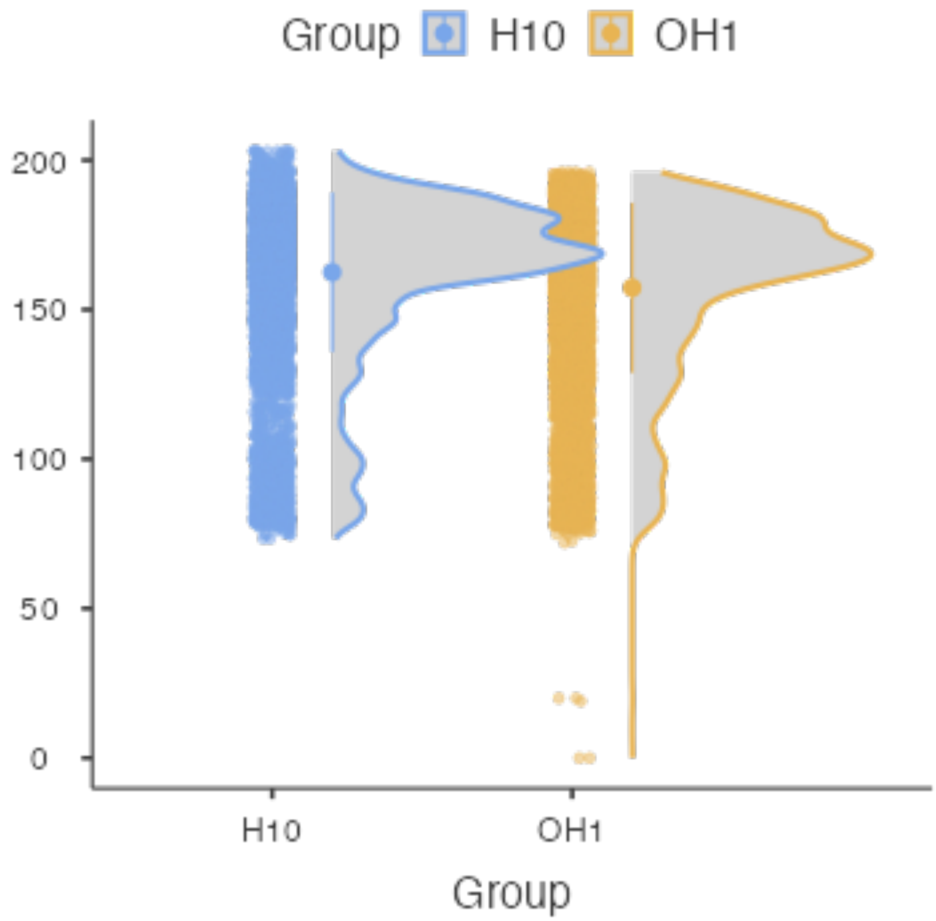

Supplementary Figure S47. Polar OH1 5 second average (entire trail run)

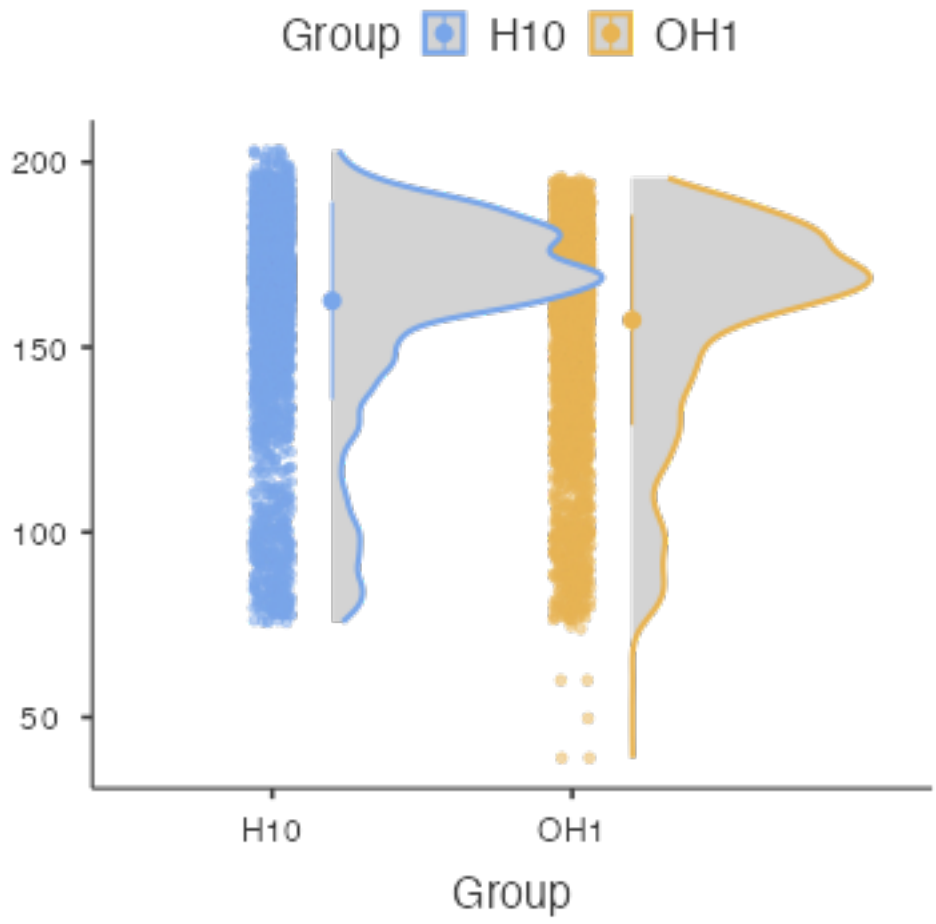

Supplementary Figure S48. Polar OH1 10 second average (entire trail run)

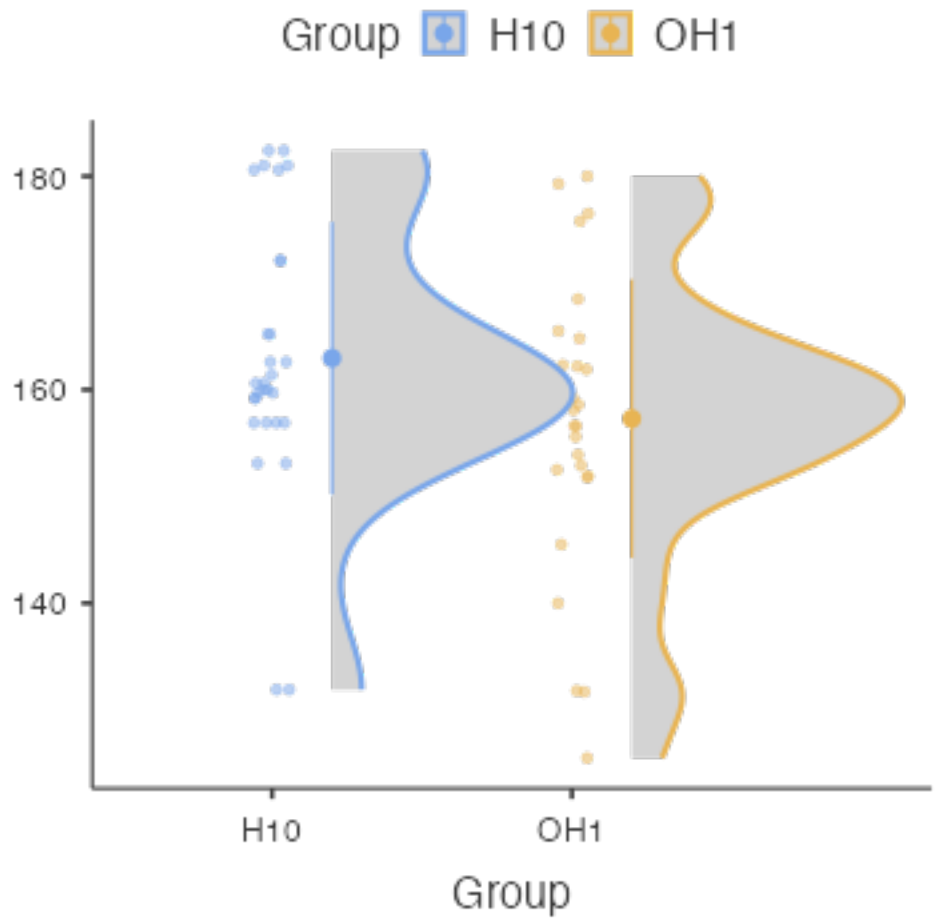

Supplementary Figure S49. Polar OH1 session average (entire trail run)

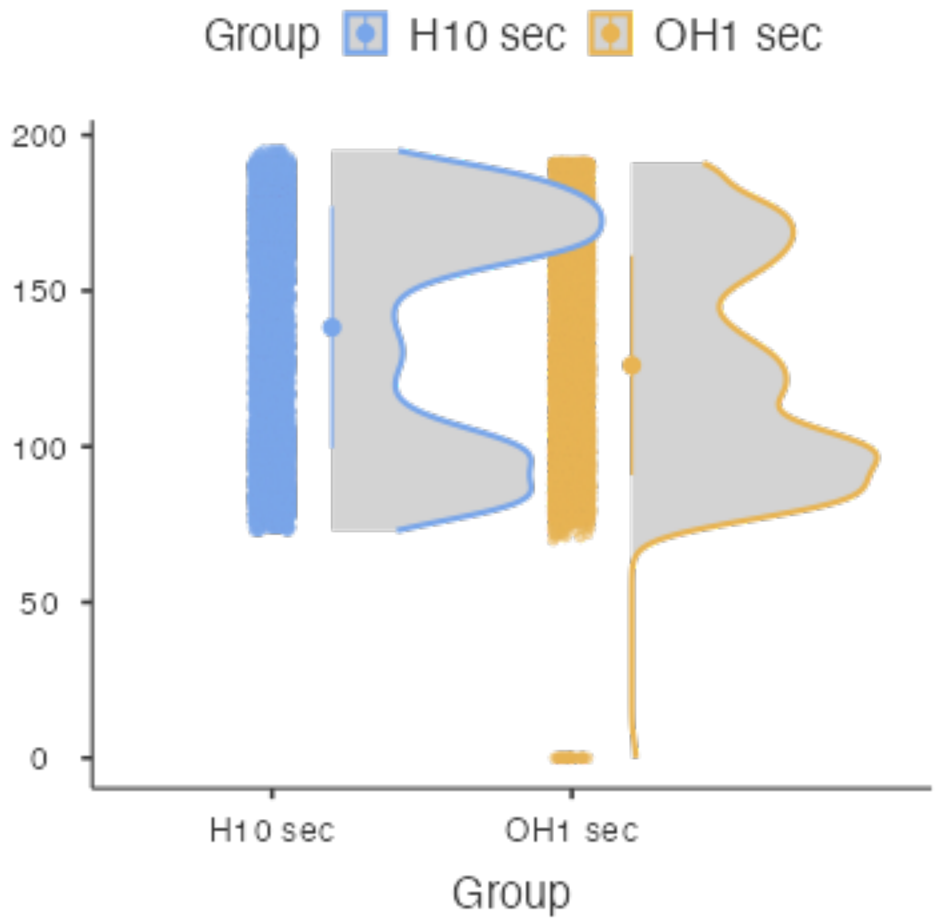

Supplementary Figure S50. Polar OH1 second-by-second (first 5 minutes only)

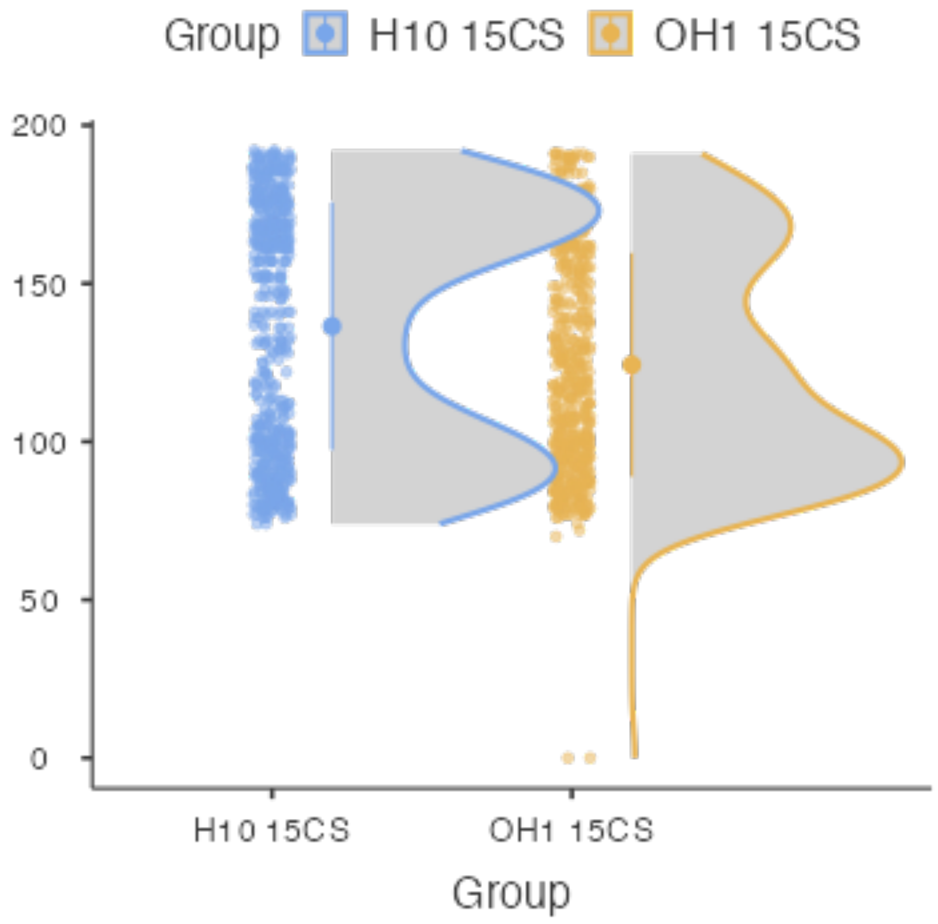

Supplementary Figure S51. Polar OH1 15 second cross-sectional (first 5 minutes only)

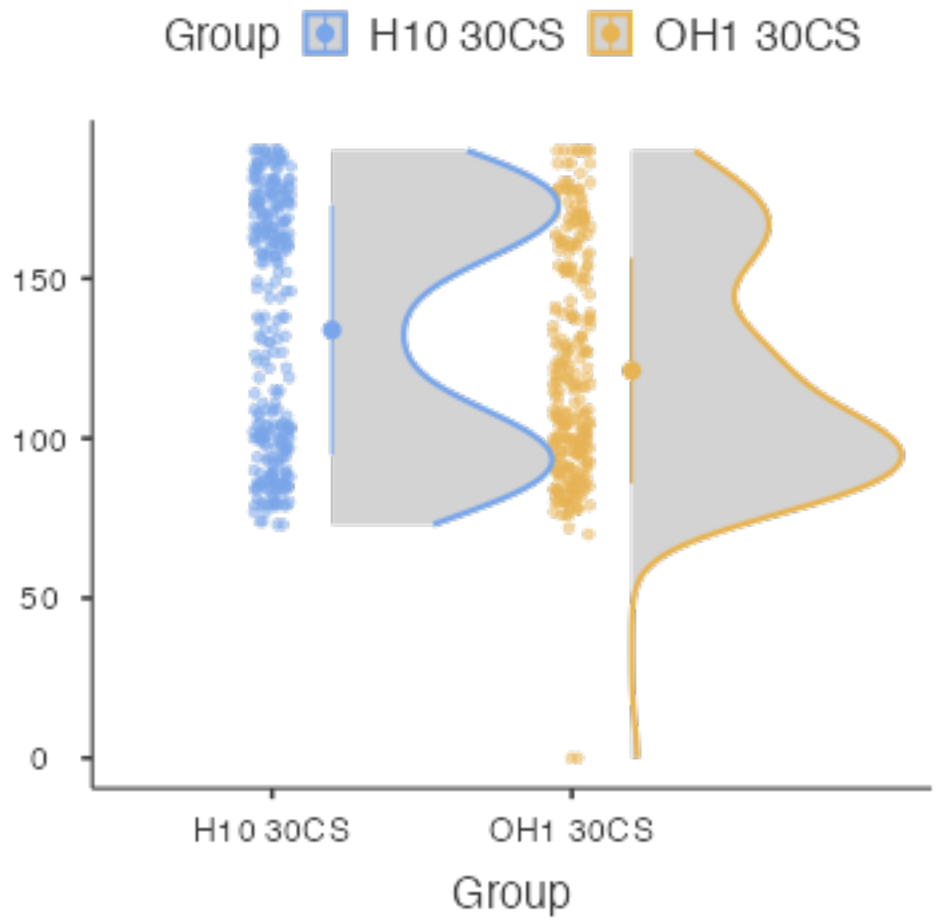

Supplementary Figure S52. Polar OH1 30 second cross-sectional (first 5 minutes only)

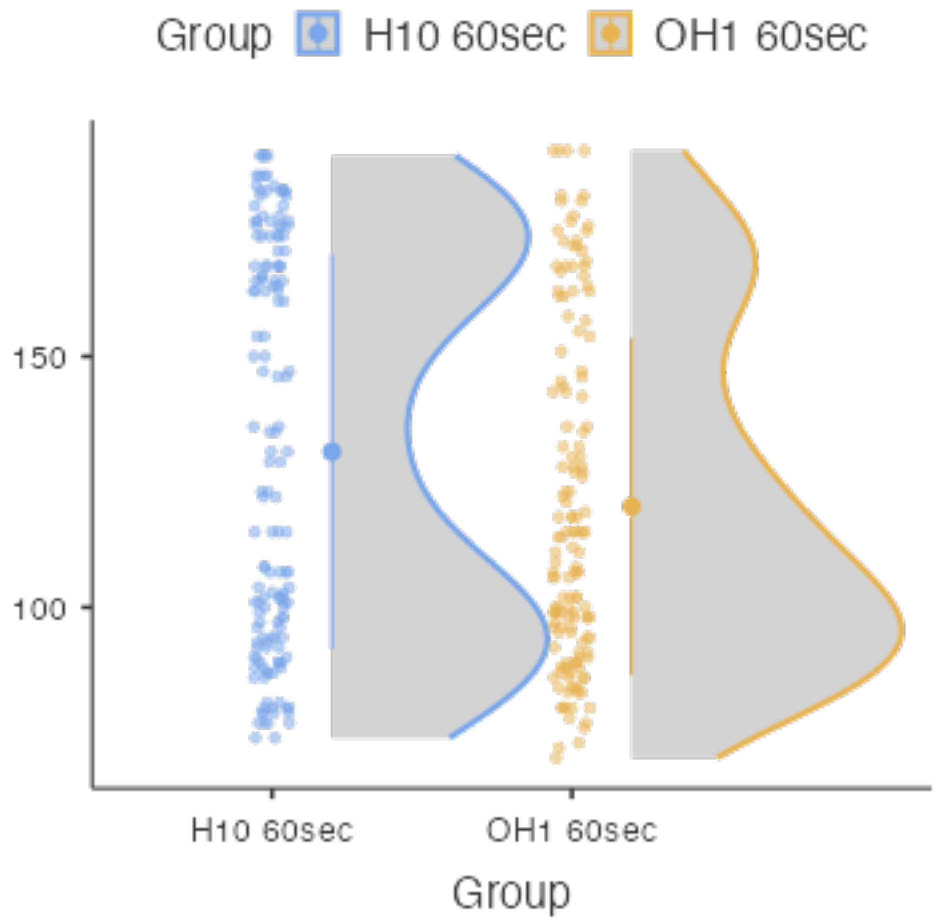

Supplementary Figure S53. Polar OH1 1 minute cross-sectional (first 5 minutes only)

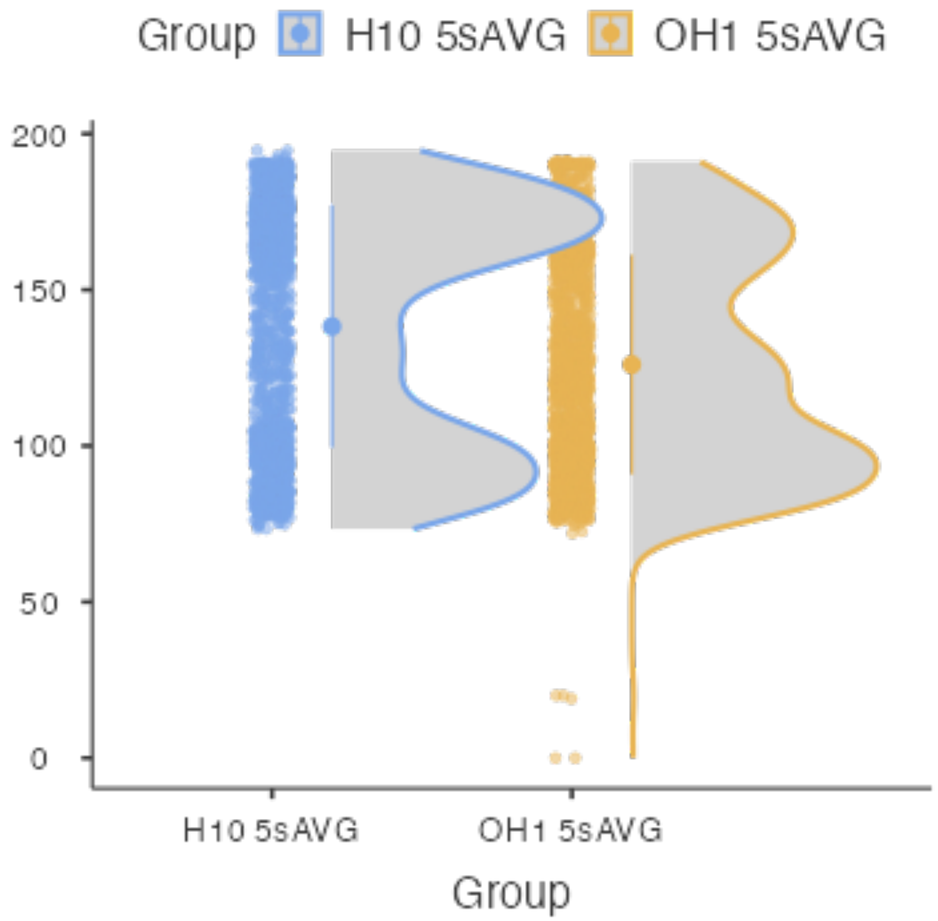

Supplementary Figure S54. Polar OH1 5 second average (first 5 minutes only)

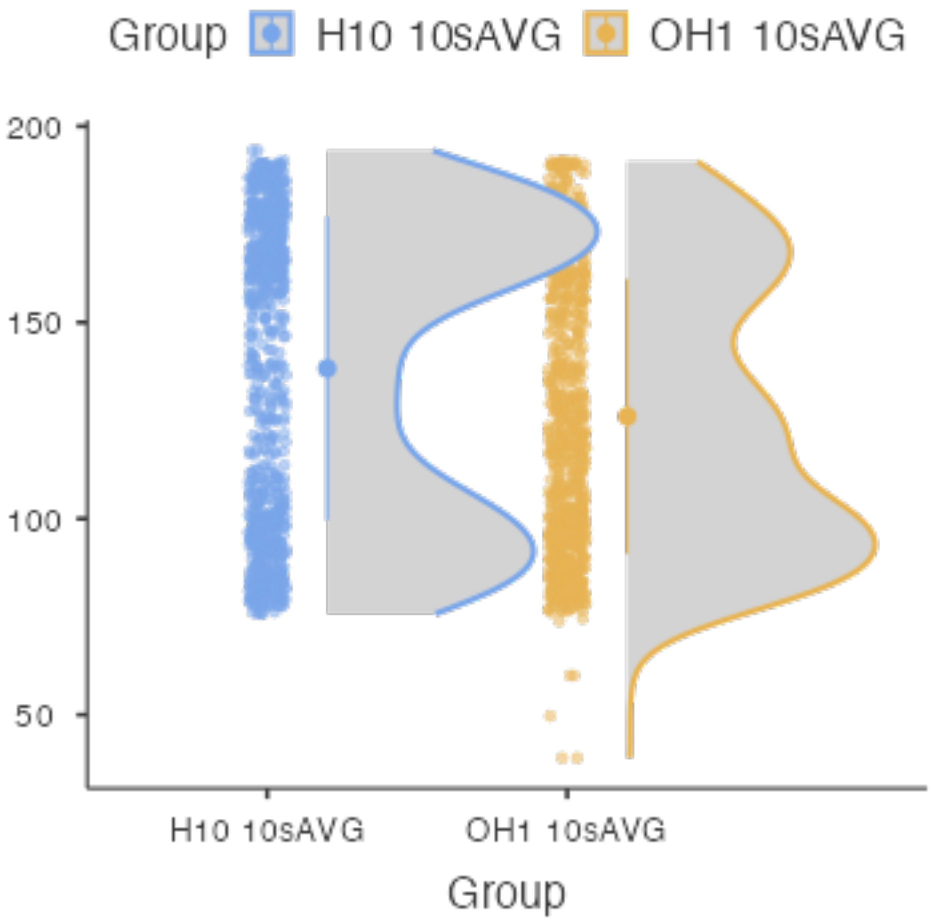

Supplementary Figure S55. Polar OH1 10 second average (first 5 minutes only)

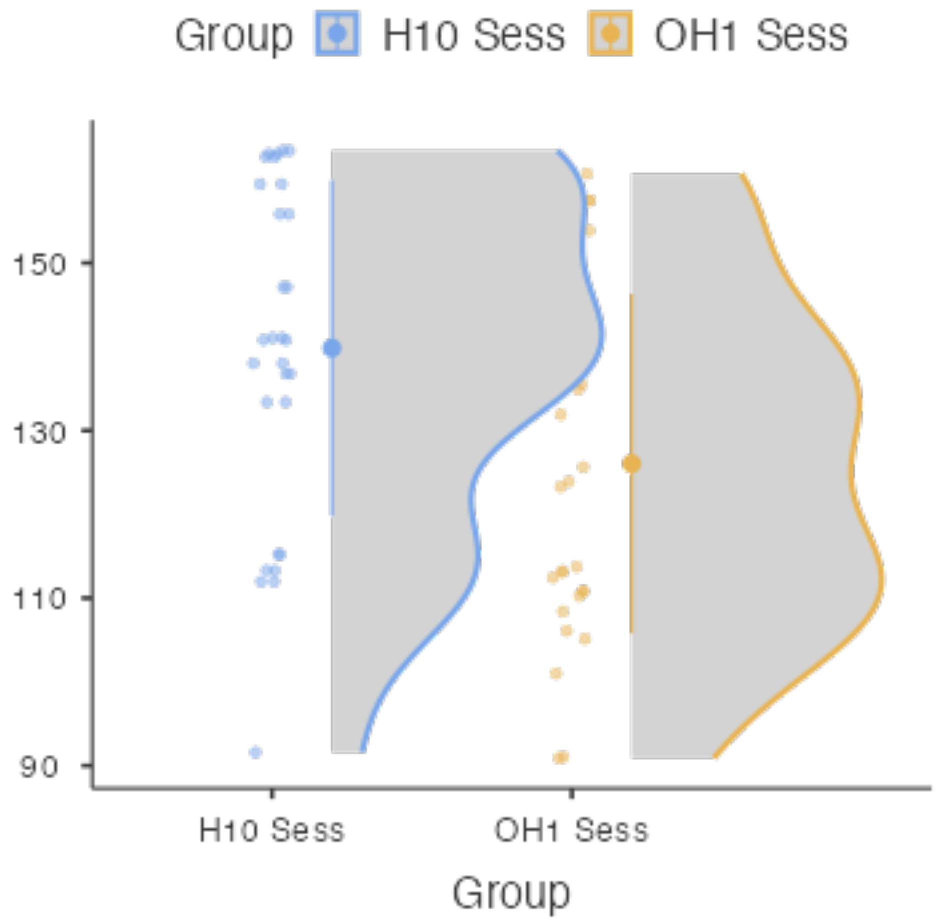

Supplementary Figure S56. Polar OH1 session average (first 5 minutes only)
